# Antistaphylococcal Activities and ADME-Related Properties of Chlorinated Arylcarbamoylnaphthalenylcarbamates

**DOI:** 10.3390/ph15060715

**Published:** 2022-06-05

**Authors:** Tomas Gonec, Dominika Pindjakova, Lucia Vrablova, Tomas Strharsky, Hana Michnova, Tereza Kauerova, Peter Kollar, Michal Oravec, Izabela Jendrzejewska, Alois Cizek, Josef Jampilek

**Affiliations:** 1 Department of Chemical Drugs, Faculty of Pharmacy, Masaryk University, Palackeho 1946/1, 61200 Brno, Czech Republic; strharsky.t@gmail.com; 2 Department of Analytical Chemistry, Faculty of Natural Sciences, Comenius University, Ilkovicova 6, 84215 Bratislava, Slovakia; pindjakova.dominika@gmail.com (D.P.); lucia.vrablova26@gmail.com (L.V.); josef.jampilek@gmail.com (J.J.); 3 Department of Infectious Diseases and Microbiology, Faculty of Veterinary Medicine, University of Veterinary Sciences Brno, Palackeho 1946/1, 61242 Brno, Czech Republic; michnova.hana@gmail.com (H.M.); cizeka@vfu.cz (A.C.); 4 Department of Pharmacology and Toxicology, Faculty of Pharmacy, Masaryk University, Palackeho 1946/1, 61200 Brno, Czech Republic; kauerovat@pharm.muni.cz; 5 Global Change Research Institute CAS, Belidla 986/4a, 60300 Brno, Czech Republic; oravec.m@czechglobe.cz; 6 Institute of Chemistry, University of Silesia, Bankowa 12, 40007 Katowice, Poland; izabela.jendrzejewska@us.edu.pl; 7 Department of Chemical Biology, Faculty of Science, Palacky University Olomouc, Slechtitelu 27, 78371 Olomouc, Czech Republic

**Keywords:** hydroxynaphthalenes, carbamates, antistaphylococcal activity, cytotoxicity, lipophilicity, structure–activity relationships

## Abstract

Pattern 1-hydroxy-*N*-(2,4,5-trichlorophenyl)-2-naphthamide and the thirteen original carbamates derived from it were prepared and characterized. All the compounds were tested against *Staphylococcus aureus* ATCC 29213 as a reference and quality control strain and in addition against three clinical isolates of methicillin-resistant *S. aureus* (MRSA). Moreover, the compounds were evaluated against *Enterococcus faecalis* ATCC 29212, and preliminary in vitro cytotoxicity of the compounds was assessed using the human monocytic leukemia cell line (THP-1). The lipophilicity of the prepared compounds was experimentally determined and correlated with biological activity. While pattern anilide had no antibacterial activity, the prepared carbamates demonstrated high antistaphylococcal activity comparable to the used standards (ampicillin and ciprofloxacin), which unfortunately were ineffective against *E. feacalis*. 2-[(2,4,5-Trichlorophenyl)carba- moyl]naphthalen-1-yl ethylcarbamate (**2**) and 2-[(2,4,5-trichlorophenyl)carbamoyl]naphthalen-1-yl butylcarbamate (**4**) expressed the nanomolar minimum inhibitory concentrations (MICs 0.018–0.064 μM) against *S. aureus* and at least two other MRSA isolates. Microbicidal effects based on the minimum bactericidal concentrations (MBCs) against all the tested staphylococci were found for nine carbamates, while 2-[(2,4,5-trichlorophenyl)carbamoyl]naphthalen-1-yl heptylcarbamate (**7**) and 2-[(2,4,5-trichlorophenyl)carbamoyl]naphthalen-1-yl (4-phenylbutyl)carbamate (**14**) demonstrated MBCs in the range of 0.124–0.461 μM. The selectivity index (SI) for most investigated carbamates was >20 and for some derivatives even >100. The performed tests did not show an effect on the damage to the bacterial membrane, while the compounds were able to inhibit the respiratory chain of *S. aureus*.

## 1. Introduction

*Staphylococcus aureus*, discovered by Scottish surgeon A. Ogston in 1880 and named by the German bacteriologist F. Rosenbach in 1884, remains one of the most common and resistant Gram-positive bacteria and thus represents a global health problem [1,2]. It causes a variety of infections: from mild skin and soft tissues inflammations to life-threatening sepsis associated with organ failure, toxic shock syndrome, and necrotizing pneumonia [2,3,4]. The high pathogenicity of *S. aureus* is due to the production of toxins that cause the destruction of the patient’s tissue. In about a third of the human population, it is naturally present on the skin and mucous membranes. The transfer of antibiotic resistance genes (on plasmids) between *S. aureus* strains is most often performed by transduction. Approximately 90% of staphylococci are resistant to common penicillin, so β-lactamase-resistant penicillins (such as oxacillin, methicillin, and cloxacillin) must be used. β-Lactamase is an enzyme that cleaves the β-lactam ring, which is an essential structural condition for the activity of these antibiotics. Aminopenicillins (ampicillin and amoxicipin) with β-lactamase inhibitors (most often clavulanic acid, sulbactam, and tazobactam) can also be used to treat staphylococcal infections [3,4,5]. Furthermore, macrolides, cephalosporins of the I and IV generations, aminoglycosides, lincosamides, tetracyclines, glycopeptides, and fluoroquinolones can be used for treatment. Mupirocin or chloramphenicol can be used topically [3,4].

In 1961, shortly after the introduction of methicillin into clinical practice, methicillin-resistant strains of *S. aureus* (MRSA) were found. Since then, their percentages in *S. aureus* populations have increased and are currently a major health problem worldwide in the treatment of staphylococcal infections. Methicillin resistance (in fact to all β-lactam antibiotics) is provided by the *mecA* gene, which encodes an alternative transpeptidase (penicillin binding protein 2a or 2’), which is the enzyme responsible for the synthesis of the bacterial cell wall. This alternative transpeptidase is not inactivated by antibiotics such as penicillin or methicillin, and the bacteria is therefore resistant to these antibiotics. In addition to the methicillin-resistance gene, these strains usually carry other genes providing resistance to other antibiotics (e.g., tetracycline and vancomycin), which poses a serious treatment problem [3,5,6,7]. Thus, *S. aureus* is one of the major pathogens for antimicrobial resistance-related deaths, and the occurrence of antibiotic-resistant strains, such as MRSA, vancomycin-resistant, or linezolid-resistant *S. aureus*, is a worldwide problem in clinical medicine. It should be added that, despite many efforts, no vaccine against *S. aureus* has been developed [4]. MRSA infection is one of the leading causes of nosocomial infections and is commonly associated with significant morbidity, mortality, length of hospital stay, and treatment costs. The reported incidence of MRSA infection ranges from 7% to 60%. A higher incidence of MRSA infection is observed in healthcare professionals. Natural colonization of MRSA increases the risk of infection and infectious strains correspond to colonizing strains in up to 50–80% of cases. MRSA can cause a number of organ-specific infections, the most common being skin and subcutaneous tissues, followed by invasive infections such as endocarditis, osteomyelitis, meningitis, pneumonia, lung abscess, and empyema [3,4,8,9].

**Scheme 1:**
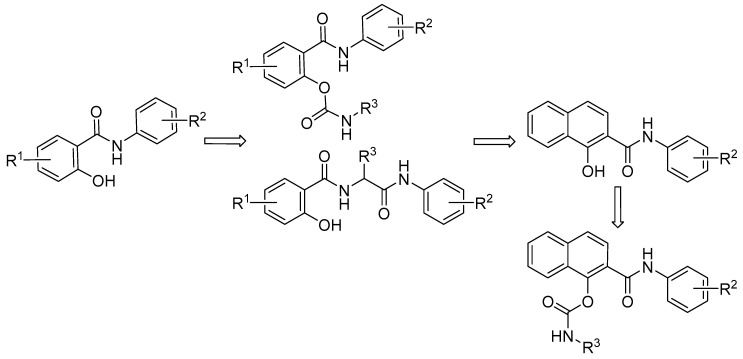
Design of the investigated carbamates based on structural analogy/bioisosterism to salicylanilide derivatives.

Thus, MRSA infections continue to be a challenge in the design of new active agents. Based on long-standing interest in anti-infective agents, small molecules structurally derived from salicylanilide and its carbamates [10,11,12,13,14,15,16,17] have been designed as naphthalene analogues of salicylanilides [18,19,20,21,22], see Scheme 1. Some of these molecules were able to effectively inhibit *S. aureus* [13,14,19,21]. All these small entities are characterized by the presence of both amide and carbamate bonds. Both functional groups cause the molecules to be able to bind to biological targets [23,24,25] and act as multitarget compounds; i.e., they are able to inhibit the growth of microorganisms by acting on several different targets simultaneously and thus reduce the risk of resistance [26,27,28,29,30]. The compounds investigated in this paper were derived from recently described 1-hydroxynaphthalene- 2-carboxanilides [18,20,21,31]. As carbamates or compounds with two amide groups tend to have stronger antibacterial potential [10,14,15,16,17], a series of carbamates was prepared from the antibacterial-ineffective, and to human cells non-toxic, 1-hydroxy-*N*-(2,4,5-trichlorophenyl)-2-naphthamide (**1**), in order to increase antimicrobial efficacy. This study describes the microwave synthesis of the starting anilide **1**, the subsequent preparation of carbamates, the investigation of ADME-related properties, and the antistaphylococcal activity.

## 2. Results and Discussion

### 2.1. Chemistry

**Scheme 2:**
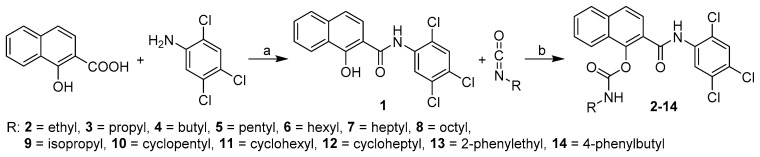
Synthesis of 2-[(2,4,5-trichlorophenyl)carbamoyl]naphthalen-1-yl carbamates **2**–**14**. Reagents and conditions: (**a**) PCl_3_, chlorobenzene, MW; (**b**) TEA, acetonitrile, ambient temperature.

The synthesis of the target carbamates was performed in two steps (Scheme 2). First, 1-hydroxy-*N*-(2,4,5-trichlorophenyl)-2-naphthamide (**1**) was prepared by the microwave-assisted method using phosphorus trichloride in dry chlorobenzene from 1-hydroxynaphthalene-2-carboxylic acid and 2,4,5-trichloroaniline [31]. In the second step, the triethylamine-activated phenolic group was reacted with the appropriate isocyanate in acetonitrile to provide a series of final 2-[(2,4,5-trichlorophenyl)carbamoyl]- naphthalen-1-yl alkyl-, cycloalkyl-, and arylalkyl-carbamates **2**–**14**, see Table 1.

The compounds were studied in detail for their physicochemical properties, as they meet the basic drug-likeness requirements, represented in particular by the Lipinski’s Rule of Five (Ro5) [32]. Lipophilicity was measured by reversed-phase high performance liquid chromatography (RP-HPLC) under isocratic conditions with methanol as the organic modifier in the aqueous mobile phase and the logarithm of the capacity factor *k* was calculated, which is used as the lipophilicity index converted to the log *P* scale [33,34]. In addition to the log *k* values, the logarithm of the distribution coefficient *D*_pH_ at pH 6.5 and 7.4 was determined to clarify the behavior of the compounds under physiological conditions and possible ionization [33,35]. Instead of water, a buffer of suitable pH was used as part of the mobile phase. In addition to the above-mentioned experimental methods, commercially available ChemBioDraw Ultra 13.0 and ACD/Percepta ver. 2012 programs were used to calculate the lipophilicity values of all the prepared compounds. All the results are shown in Table 1. In addition to lipophilicity, other parameters discussed in Ro5, such as molecular weight, number of H-bond donors/acceptors, as well as parameters not reported in Ro5, but characterizing the physicochemical properties of the prepared molecules, such as number of rotatable bonds, were predicted using ACD/Percepta. Moreover, for each R substituent, bulkiness expressed as molar volume (MV [cm^3^]), a parameter describing the tail length/branching of each carbamates, was calculated. All these parameters are listed in Table 1.

**Figure 1:**
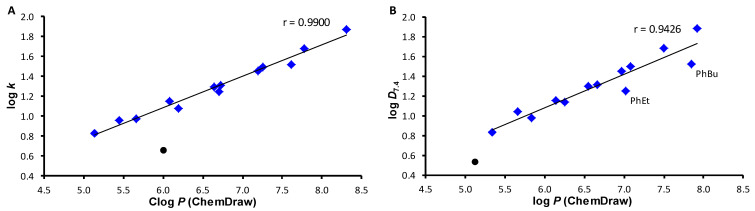
Comparison of the experimentally determined values of log *k* (**A**) and log *D*_7.4_ (**B**) with the predicted Clog *P* (ChemDrawUltra) and log *P* (ChemDrawUltra) values of all prepared compounds **1**–**14** (black circle = anilide **1**, blue rhombus = carbamates **2**–**14**).

From the experimental lipophilicity results, it is evident that the starting anilide **1** has the lowest values of log *k* and log *D*. (Note that this fact was correctly calculated only by log *P* from ACD/Percepta. ChemDrawUltra predicted ethyl carbamate **2** to be the least lipophilic.) Within the series of linear tail derivatives **2**–**8**, lipophilicity increases linearly from the ethyl (ca. 0.83) to octyl (ca. 1.87), which shows the highest lipophilicity of all carbamates. Within cyclic derivatives **10**–**13**, lipophilicity increases from cyclopentyl (ca. 1.15) to cycloheptyl (ca. 1.45). Logically, the phenylethyl derivative has a lower lipophilicity than the phenylbutyl derivative (**14**, ca. 1.25 and **15** ca. 1.52, respectively). The lipophilicity of the isopropyl derivative **9** is slightly lower (insignificantly different) from the propyl derivative **3**. The lipophilicity of cyclic derivatives **10**–**13** ranges from the level of aliphatic derivatives from butyl to hexyl carbamates **4**–**6**. The lipophilicity of the phenylalkyl carbamates **13** and **14** is at the level of pentyl/hexyl **5**/**6** and cyclohexyl/cycloheptyl **11**/**12** derivatives, respectively. It is important to note that all experimental lipophilicity values correlated with each other in the interval of the correlation coefficients r from 0.9908 to 0.9974 (*n* = 13), as shown in the graphs of Figure S1. Deviations between the log *k*/log *D* values were most evident in the parent anilide **1**, where with increasing pH, lipophilicity decreased by almost 20% (log *k* = 0.6600, log *D*_7.4_ = 0.5384), which may indicate the ability of the phenolic hydroxyl to be protonated in the basic pH medium. No significant changes in the experimental lipophilicity values were found for any of the carbamates **2**–**14**. The correlation between experimental and predicted lipophilicity values in the carbamate series **2**–**14** (*n* = 13) was the most pronounced for Clog *P* (ChemDrawUltra, ranged from 0.9826 to 0.9900), followed by log *P* (ACD/Percepta, ranged from 0.9585 to 0.9631), and the worst correlation was observed for log *P* (ChemDrawUltra, ranged from 0.9426 to 0.9505). The graphs are shown in Figures S2–S4. In general, the log *k* values correlated best with the predicted data, and log *D*_7.4_ the worst; the best and worst correlations are shown in Figure 1. The largest deviations were found for phenylalkyl derivatives **13** and **14** (for log *P* values obtained by ChemDrawUltra), smaller for cycloheptyl (for log *P* values obtained by ACD/Percepta).

Based on the lipophilicity values given in Table 1, it can be stated that even the starting anilide no longer meets the Ro5 recommendation of log *P* < 5. On the other hand, there is a chance that the compounds will penetrate better through the bacterial wall. Likewise, heptyl, octyl, cycloheptyl, and both fenylalkyl derivatives have slightly higher molecular weights than recommended. In the other parameters, the compounds meet the Ro5 recommendations. For a more detailed characterization of the carbamate tails, Table 1 shows their bulkiness expressed as molar volumes (MV (cm^3^)) of the individual carbamate substituents R. Logically, the ethyl chain (compound **2**) is the least bulky, while octyl and phenylbutyl substituents (compounds **8**, **14**) are the bulkiest. Previous studies that focused on the physicochemical and biological properties of carbamates (or, in general, agents with a polar head and a lipophilic tail) have found that the ability of these molecules to disrupt the integrity of biological membranes is extremely important for any of their biological activities [14,19,20,36,37,38,39]. The selection of a suitable chain length is extremely important and difficult for the ability of the agent to act as a surfactant, because tail length subsequently strongly affects the other related physicochemical properties of the compound. Thus, the investigated series of carbamates is characterized by a wide structural spectrum of tails. In general, the longer the lipophilic tail, the better the membrane-damaging properties of the compound. On the other hand, it is necessary to take into account the so-called cut-off effect, which is a non-linear dependence of biological activity on the molecular parameter of the surfactant, such as the length of the alkyl chain. This means that the activity only increases to a certain tail length and then decreases. The cut-off effect of the membrane active compounds (which penetrate to the membrane and disrupt its architecture) has been well documented [14,19,20,36,37,38,39,40,41,42,43,44].

### 2.2. In Vitro Antistaphylococcal Activity

All the investigated compounds were screened for in vitro antibacterial activity against the reference and quality control strain *Staphylococcus aureus* ATCC 29213 and clinical isolates of methicillin-resistant *S. aureus* (MRSA) 63718, SA 630, and SA 3202 [14]. Activities are expressed as the minimum inhibitory concentrations (MICs), see Table 2. In addition to the MICs, the minimum bactericidal concentrations (MBCs) were also determined. To establish that a compound demonstrates a bactericidal effect against a particular tested strain, it must meet the condition MIC/MBC ≤ 4 [14,45]. MBC values that meet this requirement, i.e., the compound is bactericidal, are indicated in bold in Table 2.

It can be stated that, except for starting anilide **1**, the all the compounds demonstrated high antistaphylococcal activities against both collection *S. aureus* and MRSA isolates, which significantly exceeded the activities of the clinically used drugs ampicillin and ciprofloxcin. Overall, ethyl and butyl carbamates **2** and **4** showed the lowest MIC (nanomolar) values against *S. aureus* and at least two other MRSA isolates, and ethyl **2**, butyl **4**, and phenylethyl **13** derivatives showed the lowest average MICs against all the tested staphylococci. Nevertheless, it can be stated that the MICs against the MRSA isolates were, in fact, comparable with the MIC values observed against methicillin-susceptible *S. aureus* ATCC 29213; it could be assumed that the presence of the above-mentioned *mecA* gene [46] did not affect the activity of these compounds. Microbicidal effects meeting the condition MIC/MBC ≤ 4 for all the tested staphylococci were found for propyl **3**, pentyl–octyl **5**–**8**, cyclopentyl–cycloheptyl **10**–**12,** and phenylbutyl **14** carbamates, while derivatives **7** and **14** demonstrated MBCs <0.5 μM.

Encouraged by these results, all the compounds were also evaluated on their anti-enterococcal activity, but with very unsatisfactory results. MIC values against reference strain *Enterococcus faecalis* ATCC 29212 are given in Table 2. Only ethyl carbamate **2** showed medium activity, heptyl and cyclohexyl carbamates **7** and **11** demonstrated moderate activity, and the rest of the compounds were completely inactive. A comparison of these highly potent antistaphylococcal agents with moderate or no activity against *E. feacalis* suggests that the mechanism of action against staphylococci may be different from that of enterococci. An explanation for the observed differences in the susceptibility of staphylococci and enterococci to the tested carbamates can be found in the naturally much greater resistance of enterococci to disinfection procedures [47,48]. Recent functional genomic studies [49,50] identified unique properties of enterococci that allow them to survive exposure to antibiotics.

As mentioned above, the MICs of the compounds against staphylococci are similar; thus, it can be speculated concerning the specific activity against *Staphylococcus* spp. An attempt was made to reveal the mechanism of action of these compounds, and therefore a standard MTT test was performed with the most active compounds. The MTT assay can be used to assess cell growth by measuring respiration. The MTT measured viability of bacterial cells less than 70% after exposure to the MIC values for each tested compound is considered as a positive result of this assay. This low level of cell viability indicates inhibition of cell growth by inhibition of respiration [19,20,51,52]. It can be concluded that none of the evaluated compounds showed a decrease in viability <70% at its MIC value, which suggests that the main mechanism of action is not inhibition of the respiratory chain, although they are able to significantly affect it, compared to, e.g., ciprofloxacin. The lowest multiples of the MIC values by which inhibition of *S. aureus* ATCC 29213 viability (%) greater than 70% was achieved are given in Table 3.

**Figure 2:**
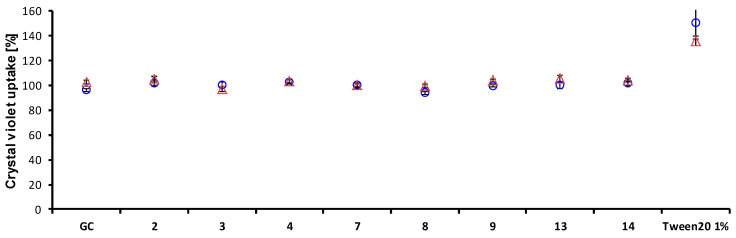
Uptake of crystal violet by *S. aureus* and MRSA2 (blue circle = *S. aureus* ATCC 29213, green tringle = clinical isolates of methicillin-resistant *S. aureus* SA 630, GC = growth control).

As mentioned above, the bactericidal activity of the carbamates was expected to be associated with their ability to disrupt the bacterial membranes. Thus, an alteration in the membrane permeability of *S. aureus* ATCC 29123 and MRSA SA 630 was detected by a hydrophobic crystal violet dye assay [53,54]. Clinical isolate MRSA2 was selected as the most resistant (bactericidally least sensitive) to the evaluated carbamates. The bacterial suspensions were treated by compounds **2**–**4**, **7**–**9**, **13**, and **14** (4× MIC) for 1 h. The uptake of crystal violet was expressed as a percentage compared to the original crystal violet solution. The results are shown in Figure 2. The test compounds did not affect plasma membrane permeability of either *S. aureus* or MRSA, because the percentage absorption of crystal violet was comparable to the growth control and disproportionately lower than the 1% Tween 20 solution used as the positive control. Based on these results, it could be assumed that an increase in the membrane permeability was not the main mechanism of action of these compounds.

### 2.3. In Vitro Cell Viability Assay

Preliminary in vitro cytotoxicity screening of the investigated compounds was performed using human monocytic leukemia THP-1 cells, see Table 2. The ability to inhibit the human cancer cell viability (IC_50_) of the compounds was compared to camptothecin toxicity (0.16 ± 0.07 μM). The cytotoxicity of the parent anilide **1** was found to be insignificant (IC_50_ >30 μM), while the ability of cell viability inhibition of carbamates **2**–**14** was in the range of IC_50_ 2.61–3.44 μM. However, these values are up to two orders of magnitude higher than their significant antistaphyloccocal concentrations. This fact is also taken into account in the calculated selectivity index (SI), Table 2, where for only three compounds the SI is <20, while for the rest of the agents SI is >20, and for some derivatives even 100 and more. Thus, based on all these observations, it can be concluded that all the tested derivatives can be considered as in vitro non-toxic compounds for the subsequent design of new therapeutic agents.

### 2.4. Structure–Activity Relationships

**Figure 3:**
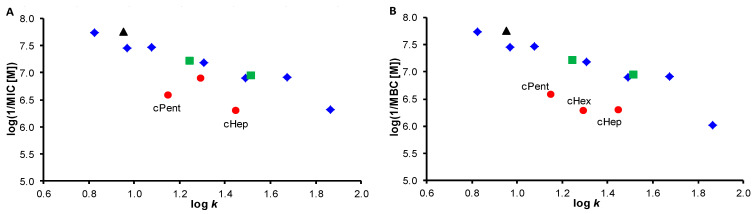
Dependences of in vitro bacteriostatic log(1/MIC (M)) (**A**) and bactericidal log(1/MBC (M)) (**B**) activity against *S. aureus* ATCC 29213 on lipophilicity expressed as log *k* of the studied compounds (red circle = cycloalkyls of **10**–**12**, green square = phenylalkyl tails of **13**, **14**, blue rhombus = aliphatic alkyl tails of **2**–**8**, black triangle = isopropyl chain **9**).

**Figure 4:**
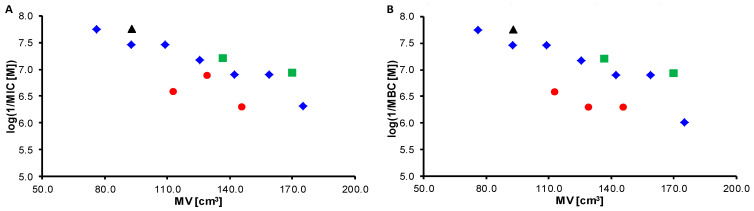
Dependences of in vitro bacteriostatic log(1/MIC (M)) (**A**) and bactericidal log(1/MBC (M)) (**B**) activity against *S. aureus* ATCC 29213 on bulkiness of individual tails expressed as the molar volume of substituents R (red circle = cycloalkyls of **10**–**12**, green square = phenylalkyl tails of **13**, **14**, blue rhombus = aliphatic alkyl tails of **2**–**8**, black triangle = isopropyl chain **9**).

As mentioned above, in terms of Ro5, all compounds have a higher lipophilicity than recommended and some derivatives have slightly higher molecular weights. Based on the values from Table 1 and the antistaphylococcal data from Table 2, the dependences of the activities against *S. aureus*, expressed as log(1/MIC) and log(1/MBC), on lipophilicity (log *k*) (Figure 3) were created. Both graphs show a clear trend where activity decreases with increasing lipophilicity. Alkyl/phenylalkyl derivatives are clearly separated from cycloalkyl carbamates **10**–**12** in Figure 3B, which illustrates the dependence of the microbicidal effect on lipophilicity. No significant differences are seen in the graphs of Figure 4, where the dependences of the activities against *S. aureus* on bulkiness of the carbamate tails, are illustrated. Figure 3B and Figure 4B (the dependences of cidal activities on physicochemical parameters) show a significant bounce of the octyl chain and low activity of octyl carbamate **8** compared to the other derivatives, which is probably related to the above-mentioned cut-off effect [36,37] and “over-bulkiness/over-length” of this tail. It should be noted that the dependences of activity expressed as log(1/MIC) shown in Figure 3A and Figure 4A against all MRSA isolates have only a slightly different course and therefore are not shown. The dependences of the bactericidal activities (log(1/MBC)) against SA 3202 (MRSA3) are also very close to the graphs in Figure 3B and Figure 4B and are not shown.

**Figure 5:**
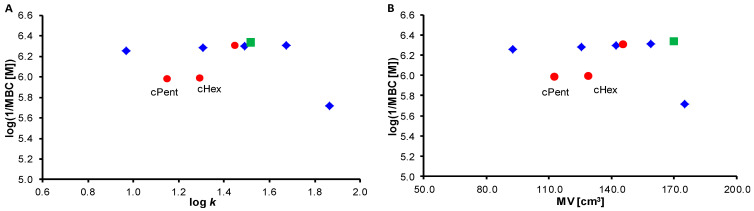
Relationships between in vitro bactericidal activity against MRSA SA 630 log(1/MBC (M)) and lipophilicity expressed as log *k* of the studied compounds (**A**), and bulkiness of the individual tails expressed as the molar volume of substituents R (**B**) (red circle = cycloalkyls of **10**–**12**, green square = phenylbutyl **14**, blue rhombus = aliphatic alkyl tails of **3**, **5**–**8**).

The dependences of bactericidal activity expressed as log(1/MBC) on the lipophilicity and bulkiness of the carbamate tails against isolates MRSA 63718 and MRSA SA 630 (MRSA1 and MRSA2) have completely different trends than above discussed. As MRSA SA 630 showed the least amount of compounds with bactericidal activity (bold values in Table 2), the dependences are demonstrated on this isolate. Based on these data, it can be stated that the compounds with medium or moderate MIC activity showed mainly bactericidal activity. From the dependence of log(1/MBC) on lipophilicity (Figure 5A), it can be stated that the activity increases with lipophilicity to the value of log *k* approx. 1.7 (heptyl derivative **7**), then decreases sharply to octyl carbamate **8** with the lowest MBC value. A similar trend can be found in the relationship of bactericidal activity on bulkiness (cidal activity increases up to phenylbutyl derivative **14** with MV ca. 170 cm^3^), then subsequently decreases sharply to carbamate **8** with the highest bulkiness and the highest lipophilicity, see Figure 5B. Based on the obtained results, it is evident that the bactericidal activity of the studied compounds against MRSA SA 630 and MRSA 63718 is connected to the presence of a longer alkyl chain in the molecule. It can only be speculated whether the different courses of dependences of bactericidal activities against *S. aureus* ATCC 29213/MRSA SA 3202 (MRSA3) from isolates MRSA1/MRSA2 could be due to their differences in the composition of bacterial membranes [55,56,57,58]. The effect of changing the length of the hydrocarbon tail affecting the extent of antimicrobial activity of the compounds has been discussed (e.g., [36,37,39,40,41,42,43,44,59,60]). The decrease in biological activity at a higher chain length as a cut-off effect is discussed above.

## 3. Materials and Methods

### 3.1. General Methods

All reagents were purchased from Merck (Sigma-Aldrich, St. Louis, MO, USA) and Alfa (Alfa-Aesar, Ward Hill, MA, USA). Microwave-assisted reactions were performed using a StartSYNTH microwave lab station (Milestone, Sorisole, Italy). The melting points were determined on a Kofler hot-plate apparatus HMK (Franz Kustner Nacht KG, Dresden, Germany) and are uncorrected. Infrared (IR) spectra were recorded on an ATR diamond iD7 for Nicolet™ Impact 410 Fourier-transform IR spectrometer (Thermo Scientific, West Palm Beach, FL, USA). The spectra were obtained by the accumulation of 64 scans with a 2 cm^−1^ resolution in the region of 4000–650 cm^−1^. All ^1^H- and ^13^C-NMR spectra were recorded on an JEOL ECZR 400 MHz NMR spectrometer (400 MHz for ^1^H and 100 MHz for ^13^C, Jeol, Tokyo, Japan) in dimethyl sulfoxide-*d*_6_ (DMSO-*d*_6_). ^1^H and ^13^C chemical shifts (δ) are reported in ppm. High-resolution mass spectra were measured using a high-performance liquid chromatograph Dionex UltiMate^®^ 3000 (Thermo Scientific, West Palm Beach, FL, USA) coupled with an LTQ Orbitrap XL^TM^ Hybrid Ion Trap-Orbitrap Fourier Transform Mass Spectrometer (Thermo Scientific) equipped with a HESI II (heated electrospray ionization) source in the positive mode.

### 3.2. Synthesis

The synthetic pathway and characterization of 1-hydroxy-*N*-(2,4,5-trichlorophenyl)- 2-naphthamide (**1**) were described recently by Gonec et al. [31].

General procedure for synthesis of 2-[*N*-(2,4,5-trichlorophenyl)carbamoyl]naphtha- len-1-yl carbamates **2**–**14**.

Compound **1** (1.0 mmol) and triethylamine (1.1 mmol) were dissolved in dry acetonitrile (10 mL). The solution of the appropriate alkyl isocyanate (1.2 mmol) in acetonitrile (5 mL) was added in four portions within 2 h, and the reacting mixture was stirred for 24 h at ambient temperature. The solvent was evaporated under reduced pressure, and the solid residue was crystallized from methanol. The solid was collected by filtration and washed with diethyl ether to give a pure product. All the studied compounds are presented in Table 1.

*2-[N-(2,4,5-Trichlorophenyl)carbamoyl]naphthalen-1-yl ethyl carbamate* (**2**). Yield 88%; mp 190–192 °C; IR (cm^−1^): 3404, 3332, 1747, 1673, 1570, 1501, 1462, 1447, 1434, 1361, 1287, 1247, 1202, 1150, 1140, 1113, 1071, 1041, 982, 935, 923, 886, 871, 831, 818, 775, 757; ^1^H NMR (DMSO-d_6_) δ: 10.42 (s, 1H), 8.13 (s, 1H), 8.05 (d, 1H, *J* = 9.1 Hz), 8.03 (d, 1H, *J* = 8.7 Hz), 7.98–8.00 (m, 2H), 7.91 (t, 1H, *J* = 5.5 Hz), 7.63 (ddd, 1H, *J* = 8.2 Hz, *J* = 6.9 Hz, *J* = 1.4 Hz), 7.58 (ddd, 1H, *J* = 7.8 Hz, *J* = 6.9 Hz, *J* = 0.9 Hz), 7.43 (d, 1H, *J* = 8.7 Hz), 3.11 (dq, 2H, *J* = 5.5 Hz, *J* = 7.3 Hz), 1.08 (t, 3H, *J* = 7.1 Hz) (Figure S5); ^13^C NMR (DMSO-d_6_) δ: 164.69, 153.84, 145.34, 134.82, 130.76, 130.51, 130.20, 130.07, 129.86, 128.67, 128.06, 128.06, 127.76, 127.44, 126.04, 125.87, 124.71, 122.58, 35.43, 14.86 (Figure S6); HR-MS C_20_H_15_Cl_3_N_2_O_3_ [M+H]^+^ calculated 437.02210 m/z, found 437.02155 m/z.

*2-[N-(2,4,5-Trichlorophenyl)carbamoyl]naphthalen-1-yl propyl carbamate* (**3**). Yield 85%; mp 175–178 °C; IR (cm^−1^): 3402, 3363, 1748, 1671, 1570, 1501, 1457, 1433, 1360, 1286, 1261, 1200, 1164, 1139, 1105, 1071, 1043, 1012, 966, 939, 884, 856, 818, 790, 774, 756, 679; ^1^H NMR (DMSO-d_6_) δ: 10.42 (s, 1H), 8.14 (s, 1H), 8.06 (d, 1H, *J* = 9.1 Hz), 8.03 (d, 1H, *J* = 8.2 Hz), 7.98–8.00 (m, 2H), 7.93 (t, 1H, *J* = 5.7 Hz), 7.63 (ddd, 1H, *J* = 8.2 Hz, *J* = 6.9 Hz, *J* = 1.4 Hz), 7.58 (ddd, 1H, *J* = 7.8 Hz, *J* = 6.9 Hz, *J* = 0.9 Hz), 7.42 (d, 1H, *J* = 9.1 Hz), 3.01–3.06 (m, 2H), 1.43–1.52 (m, 2H), 0.86 (t, 3H, *J* = 7.5 Hz) (Figure S7); ^13^C NMR (DMSO-d_6_) δ: 164.69, 154.07, 145.37, 134.83, 130.75, 130.52, 130.19, 130.09, 129.86, 128.64, 128.07, 127.97, 127.69, 127.44, 126.05, 125.87, 124.69, 122.58, 42.34, 22.50, 11.19 (Figure S8); HR-MS C_21_H_17_Cl_3_N_2_O_3_ [M+H]^+^ calculated 451.03775 m/z, found 451.03699 m/z.

*2-[N-(2,4,5-Trichlorophenyl)carbamoyl]naphthalen-1-yl butyl carbamate* (**4**). Yield 83%; mp 178–180 °C; IR (cm^−1^): 3274, 3208, 2957, 1719, 1670, 1567, 1541, 1510, 1473, 1454, 1435, 1343, 1295, 1259, 1224, 1150, 1105, 1082, 1049, 1003, 979, 869, 815, 756, 737, 696, 676, 662; ^1^H NMR (DMSO-d_6_) δ: 10.38 (s, 1H), 8.15 (s, 1H), 8.06 (d, 1H, *J* = 9.1 Hz), 8.03 (d, 1H, *J* = 8.2 Hz), 7.98–8.00 (m, 2H), 7.91 (t, 1H, *J* = 5.5 Hz), 7.63 (ddd, 1H, *J* = 8.2 Hz, *J* = 6.9 Hz, *J* = 1.4 Hz), 7.58 (ddd, 1H, *J* = 7.8 Hz, *J* = 6.9 Hz, *J* = 0.9 Hz), 7.42 (d, 1H, *J* = 8.7 Hz), 3.05–3.09 (m, 2H), 1.38–1.46 (m, 2H), 1.24–1.33 (m, 2H), 0.84 (t, 3H, *J* = 7.3 Hz) (Figure S9); ^13^C NMR (DMSO-d_6_) δ: 164.66, 154.04, 145.37, 134.82, 130.74, 130.52, 130.19, 130.12, 129.86, 128.56, 128.06, 127.78, 127.53, 127.43, 126.05, 125.86, 124.67, 122.57, 40.24, 31.34, 19.38, 13.64 (Figure S10); HR-MS C_22_H_19_Cl_3_N_2_O_3_ [M+H]^+^ calculated 465.05340 m/z, found 465.05255 m/z.

*2-[N-(2,4,5-Trichlorophenyl)carbamoyl]naphthalen-1-yl pentyl carbamate* (**5**). Yield 90%; mp 185–186 °C; IR (cm^−1^): 3281, 3204, 2958, 2928, 719, 1672, 1566, 1538, 1511, 1472, 1453, 1341, 1294, 1257, 1221, 1149, 1122, 1082, 1045, 1017, 989, 902, 868, 814, 737, 697, 680, 663; ^1^H NMR (DMSO-d_6_) δ: 10.36 (s, 1H), 8.17 (s, 1H), 8.06 (d, 1H, *J* = 9.1 Hz), 8.03 (d, 1H, *J* = 8.2 Hz), 7.97–7.99 (m, 2H), 7.90 (t, 1H, *J* = 5.5 Hz), 7.63 (ddd, 1H, *J* = 8.2 Hz, *J* = 6.9 Hz, *J* = 1.4 Hz), 7.57 (ddd, 1H, *J* = 7.8 Hz, *J* = 6.9 Hz, *J* = 0.9 Hz), 7.42 (d, 1H, *J* = 8.7 Hz), 3.03–3.08 (m, 2H), 1.39–1.47 (m, 2H), 1.21–1.26 (m, 4H), 0.82 (t, 3H, *J* = 6.4 Hz) (Figure S11); ^13^C NMR (DMSO-d_6_) δ: 164.65, 154.03, 145.36, 134.82, 130.73, 130.53, 130.19, 130.13, 129.86, 125.54, 128.06, 128.06, 127.71, 127.44, 126.06, 125.86, 124.67, 122.57, 40.52, 28.89, 28.36, 21.04, 13.83 (Figure S12); HR-MS C_23_H_21_Cl_3_N_2_O_3_ [M+H]^+^ calculated 479.06905 m/z, found 479.06863 m/z.

*2-[N-(2,4,5-Trichlorophenyl)carbamoyl]naphthalen-1-yl hexyl carbamate* (**6**). Yield 81%; mp 160–163 °C; IR (cm^−1^): 3273, 3195, 2928, 1716, 1666, 1537, 1514, 1474, 1455, 1434, 1340, 1292, 1252, 1222, 1145, 1130, 1080, 1045, 1006, 872, 819, 756, 736, 691, 669; ^1^H NMR (DMSO-d_6_) δ: 10.35 (s, 1H), 8.17 (s, 1H), 8.06 (d, 1H, *J* = 9.1 Hz), 8.03 (d, 1H, *J* = 8.2 Hz), 7.97–7.99 (m, 2H), 7.90 (t, 1H, *J* = 5.7 Hz), 7.63 (ddd, 1H, *J* = 8.2 Hz, *J* = 6.9 Hz, *J* = 1.4 Hz), 7.57 (ddd, 1H, *J* = 7.8 Hz, *J* = 6.9 Hz, *J* = 0.9 Hz), 7.41 (d, 1H, *J* = 9.1 Hz), 3.03–3.08 (m, 2H), 1.39–1.46 (m, 2H), 1.18–1.28 (m, 6H), 0.83 (t, 3H, *J* = 6.9 Hz) (Figure S13); ^13^C NMR (DMSO-d_6_) δ: 164.64, 154.04, 145.36, 134.82, 130.72, 130.54, 130.19, 130.16, 129.87, 128.51, 128.06, 127.63, 127.44, 127.40, 126.08, 125.87, 124.67, 122.56, 40.54, 31.03, 29.20, 25.87, 22.03, 13.90 (Figure S14); HR-MS C_24_H_23_Cl_3_N_2_O_3_ [M+H]^+^ calculated 493.08470 m/z, found 493.08414 m/z.

*2-[N-(2,4,5-Trichlorophenyl)carbamoyl]naphthalen-1-yl heptyl carbamate* (**7**). Yield 71%; mp 150–153 °C; IR (cm^−1^): 3336, 3241, 3110, 1623, 1562,1505, 1461, 1452, 1403, 1361, 1314, 1270, 1235, 1195, 1156, 1136, 1074, 1041, 973, 900, 876, 863, 828, 795, 749, 725, 679; ^1^H NMR (DMSO-d_6_) δ: 10.35 (s, 1H), 8.17 (s, 1H), 8.06 (d, 1H, *J* = 8.7 Hz), 8.03 (d, 1H, *J* = 8.2 Hz), 7.98 (d, 1H, *J* = 7.8 Hz), 7.98 (s, 1H), 7.90 (t, 1H, *J* = 5.7 Hz), 7.63 (ddd, 1H, *J* = 8.2 Hz, *J* = 6.9 Hz, *J* = 1.4 Hz), 7.57 (ddd, 1H, *J* = 7.8 Hz, *J* = 6.9 Hz, *J* = 0.9 Hz), 7.41 (d, 1H, *J* = 8.7 Hz), 3.03–3.08 (m, 2H), 1.39–1.46 (m, 2H), 1.15–1.27 (m, 8H), 0.84 (t, 3H, *J* = 7.1 Hz) (Figure S15); ^13^C NMR (DMSO-d_6_) δ: 184.64, 154.05, 145.35, 134.83, 130.71, 130.54, 130.19, 130.15, 129.87, 128.51, 128.06, 128.06, 127.65, 127.44, 126.09, 125.87, 124.67, 12.57, 40.53, 31.23, 29.24, 28.50, 26.16, 22.09, 13.95 (Figure S16); HR-MS C_25_H_23_Cl_3_N_2_O_3_ [M+H]^+^ calculated 507.10035 m/z, found 507.09958 m/z.

*2-[N-(2,4,5-Trichlorophenyl)carbamoyl]naphthalen-1-yl octyl carbamate* (**8**). Yield 63%; mp 147–150 °C; IR (cm^−1^): 3194, 2923, 2852, 1712, 1656, 1567, 1511, 1473, 1454, 1432, 1360, 1297, 1276, 1258, 1215, 1157, 1143, 1076, 1040, 982, 901, 869, 826, 754, 732, 679; ^1^H NMR (DMSO-d_6_) δ: 10.35 (s, 1H), 8.17 (s, 1H), 8.06 (d, 1H, *J* = 9.1 Hz), 8.03 (d, 1H, *J* = 8.2 Hz), 7.97–7.99 (m, 2H), 7.90 (t, 1H, *J* = 5.7 Hz), 7.63 (ddd, 1H, *J* = 8.2 Hz, *J* = 6.9 Hz, *J* = 1.4 Hz), 7.57 (ddd, 1H, *J* = 7.8 Hz, *J* = 6.9 Hz, *J* = 0.9 Hz), 7.41 (d, 1H, *J* = 8.7 Hz), 3.03–3.08 (m, 2H), 1.39–1.46 (m, 2H), 1.17–1.27 (m, 10H), 0.85 (t, 3H, *J* = 6.9 Hz) (Figure S17); ^13^C NMR (DMSO-d_6_) δ: 164.65, 154.06, 145.36, 134.84, 130.71, 130.55, 130.20, 130.16, 129.88, 128.53, 128.07, 127.68, 127.45, 127.42, 126.11, 125.87, 124.68, 122.58, 40.54, 31.28, 29.25, 28.81, 28.67, 26.22, 22.12, 13.96 (Figure S18); HR-MS C_26_H_27_Cl_3_N_2_O_3_ [M+H]^+^ calculated 521.11600 m/z, found 521.11511 m/z.

*2-[N-(2,4,5-Trichlorophenyl)carbamoyl]naphthalen-1-yl isopropyl carbamate* (**9**). Yield 61%; mp 180–182 °C; IR (cm^−1^): 3256, 2976, 1721, 1660, 1565, 1508, 1455, 1359, 1322, 1284, 1250, 1227, 1176, 1145, 1079, 1061, 1022, 896, 871, 820, 774, 760, 671; ^1^H NMR (DMSO-d_6_) δ: 10.39 (s, 1H), 8.13 (s, 1H), 8.05 (d, 1H, *J* = 9.1 Hz), 8.03 (d, 1H, *J* = 8.2 Hz), 7.98–8.00 (m, 2H), 7.86 (d, 1H, *J* = 7.8 Hz), 7.63 (ddd, 1H, *J* = 8.2 Hz, *J* = 6.9 Hz, *J* = 1.4 Hz), 7.57 (ddd, 1H, *J* = 7.8 Hz, *J* = 6.9 Hz, *J* = 0.9 Hz), 7.42 (d, 1H, *J* = 9.1 Hz), 3.62–3.74 (m, 1H), 1.12 (d, 6H, *J* = 6.4 Hz) (Figure S19); ^13^C NMR (DMSO-d_6_) δ: 164.70, 153.17, 145.37, 134.84, 130.73, 130.51, 130.20, 130.08, 129.84, 128.66, 128.04, 128.03, 127.76, 127.43, 126.11, 125.85, 124.68, 122.62, 42.85, 22.38 (Figure S20); HR-MS C_21_H_17_Cl_3_N_2_O_3_ [M+H]^+^ calculated 451.03775 m/z, found 451.03662 m/z.

*2-[N-(2,4,5-Trichlorophenyl)carbamoyl]naphthalen-1-yl cyclopentyl carbamate* (**10**). Yield 78%; mp 191–193 °C; IR (cm^−1^): 3227, 2955, 1720, 1674, 1579, 1542, 1517, 1461, 1341, 1297, 1236, 1136, 1084, 1051, 1010, 877, 820, 767, 734, 709, 667; ^1^H NMR (DMSO-d_6_) δ: 10.39 (s, 1H), 8.12 (s, 1H), 8.05 (d, 1H, *J* = 9.1 Hz), 8.02 (d, 1H, *J* = 8.2 Hz), 7.98–8.00 (m, 2H), 7.95 (d, 1H, *J* = 7.3 Hz), 7.63 (ddd, 1H, *J* = 8.2 Hz, *J* = 6.9 Hz, *J* = 1.4 Hz), 7.57 (ddd, 1H, *J* = 7.8 Hz, *J* = 6.9 Hz, *J* = 0.9 Hz), 7.42 (d, 1H, *J* = 9.1 Hz), 3.82–3.90 (m, 1H), 1.17–1.83 (m, 2H), 1.61–1.68 (m, 2H), 1.46–1.52 (m, 4H) (Figure S21); ^13^C NMR (DMSO-d_6_) δ: 164.70, 153.48, 145.39, 134.83, 130.73, 130.51, 130.19, 130.08, 129.85, 128.69, 128.07, 128.04, 127.81, 127.43, 126.11, 125.85, 124.68, 122.64, 52.47, 32.14, 23.30 (Figure S22); HR-MS C_23_H_19_Cl_3_N_2_O_3_ [M+H]^+^ calculated 477.05340 m/z, found 477.05246 m/z.

*2-[N-(2,4,5-Trichlorophenyl)carbamoyl]naphthalen-1-yl cyclohexyl carbamate* (**11**). Yield 85%; mp 196–200 °C; IR (cm^−1^): 3260, 3205, 2930, 1719, 1673,1571, 1538, 1509, 1479, 1461, 1338, 1317, 1295, 1275, 1224, 1149, 1081, 1058, 1047, 1016, 876, 816, 761, 738, 713, 663; ^1^H NMR (DMSO-d_6_) δ: 10.38 (s, 1H), 8.12 (s, 1H), 8.05 (d, 1H, *J* = 9.1 Hz), 8.03 (d, 1H, *J* = 8.7 Hz), 7.98–8.00 (m, 2H), 7.88 (d, 1H, *J* = 7.8 Hz), 7.63 (ddd, 1H, *J* = 8.2 Hz, *J* = 6.9 Hz, *J* = 1.4 Hz), 7.57 (ddd, 1H, *J* = 7.8 Hz, *J* = 6.9 Hz, *J* = 0.9 Hz), 7.41 (d, 1H, *J* = 8.7 Hz), 3.30–3.35 (m, 1H), 1.67–1.84 (m, 4H), 1.53–1.58 (m, 1H), 1.07–1.29 (m, 5H) (Figure S23); ^13^C NMR (DMSO-d_6_) δ: 164.69, 153.21, 145.41, 134.85, 130.73, 130.51, 130.20, 130.11, 129.84, 128.67, 128.04, 128.04, 127.80, 127.43, 126.11, 125.85, 124.69, 122.58, 49.95, 32.50, 25.11, 24.56 (Figure S24); HR-MS C_24_H_21_Cl_3_N_2_O_3_ [M+H]^+^ calculated 491.06905 m/z, found 491.06799 m/z.

*2-[N-(2,4,5-Trichlorophenyl)carbamoyl]naphthalen-1-yl cycloheptyl carbamate* (**12**). Yield 81%; mp 196–200 °C; IR (cm^−1^): 3313, 3245, 2930, 1709, 1671, 1559, 1536, 1512, 1491, 1454, 1430, 1358, 1313, 1279, 1247, 1219, 1203, 1159, 1140, 1128, 1077, 1044, 1008, 995, 901, 881, 825, 817, 764, 753, 728, 677; ^1^H NMR (DMSO-d_6_) δ: 10.35 (s, 1H), 8.13 (s, 1H), 8.05 (d, 1H, *J* = 9.1 Hz), 8.02 (d, 1H, *J* = 8.7 Hz), 7.98–8.00 (m, 2H), 7.92 (d, 1H, *J* = 8.2 Hz), 7.63 (ddd, 1H, *J* = 8.2 Hz, *J* = 6.9 Hz, *J* = 1.4 Hz), 7.57 (ddd, 1H, *J* = 7.8 Hz, *J* = 6.9 Hz, *J* = 0.9 Hz), 7.41 (d, 1H, *J* = 8.7 Hz), 3.50–3.59 (m, 1H), 1.79–1.86 (m, 2H), 1.33–1.65 (m, 10H) (Figure S25); ^13^C NMR (DMSO-d_6_) δ: 164.70, 153.10, 145.44, 134.85, 130.72, 130.51, 130.20, 130.11, 129.85, 128.62, 128.04, 127.93, 127.70, 127.43, 126.12, 125.85, 124.67, 122.63, 52.15, 34.36, 27.80, 23.55 (Figure S26); HR-MS C_25_H_23_Cl_3_N_2_O_3_ [M+H]^+^ calculated 505.08470 m/z, found 505.08359 m/z.

*2-[N-(2,4,5-Trichlorophenyl)carbamoyl]naphthalen-1-yl 2-phenylethyl carbamate* (**13**). Yield 45%; mp 174–175 °C; IR (cm^−1^): 3404, 3317, 1749, 1672, 1572, 1504, 1447, 1434, 1362, 1289, 1254, 1210, 1141, 1099, 1071, 1042, 974, 919, 886, 833, 818, 747, 730, 702, 680; ^1^H NMR (DMSO-d_6_) δ: 10.39 (s, 1H), 8.18 (s, 1H), 8.01–8.07 (m, 3H), 7.99 (d, 1H, *J* = 7.8 Hz), 7.99 (s, 1H), 7.63 (ddd, 1H, *J* = 8.2 Hz, *J* = 6.9 Hz, *J* = 1.4 Hz), 7.58 (ddd, 1H, *J* = 7.8 Hz, *J* = 6.9 Hz, *J* = 0.9 Hz), 7.39 (d, 1H, *J* = 8.7 Hz), 7.19–7.29 (m, 5H), 3.28–3.33 (m, 2H), 2.78 (t, 2H, *J* = 7.1 Hz) (Figure S27); ^13^C NMR (DMSO-d_6_) δ: 164.63, 153.96, 145.28, 139.06, 134.79, 130.73, 130.49, 130.17, 130.05, 129.85, 128.64, 128.64, 128.52, 128.29, 128.04, 127.75, 127.42, 126.10, 125.93, 125.85, 124.66, 122.44, 42.14, 35.17 (Figure S28); HR-MS C_26_H_19_Cl_3_N_2_O_3_ [M+H]^+^ calculated 513.05340 m/z, found 513.05231 m/z.

*2-[N-(2,4,5-Trichlorophenyl)carbamoyl]naphthalen-1-yl 4-phenylbutyl carbamate* (**14**). Yield 68%; mp 180–181 °C; IR (cm^−1^): 3253, 3205, 3067, 1719, 1668, 1550, 1510, 1476, 1458, 1433, 1339, 1295, 1257, 1225, 1147, 1084, 998, 875, 818, 758, 740, 697, 676; ^1^H NMR (DMSO-d_6_) δ: 10.37 (s, 1H), 8.16 (s, 1H), 8.05 (d, 1H, *J* = 9.1 Hz), 8.02 (d, 1H, *J* = 8.2 Hz), 7.98 (d, 1H, *J* = 8.2 Hz), 7.96 (s, 1H), 7.92 (t, 1H, *J* = 5.7 Hz), 7.63 (ddd, 1H, *J* = 8.2 Hz, *J* = 6.9 Hz, *J* = 1.4 Hz), 7.57 (ddd, 1H, *J* = 7.8 Hz, *J* = 6.9 Hz, *J* = 0.9 Hz), 7.41 (d, 1H, *J* = 8.7 Hz), 7.23–7.27 (m, 2H), 7.14–7.17 (m, 3H), 3.08–3.12 (m, 2H), 2.54 (t, 2H, *J* = 7.5 Hz), 1.54–1.62 (m, 2H), 1.44–1.51 (m, 2H) (Figure S29); ^13^C NMR (DMSO-d_6_) δ: 164.64, 154.02, 145.34, 142.00, 134.80, 130.69, 130.50, 130.18, 130.08, 129.85, 128.53, 128.21, 128.15, 128.03, 127.75, 127.48, 127.41, 126.01, 125.84, 125.59, 124.67, 122.51, 40.32, 34.77, 28.86, 28.08 (Figure S30); HR-MS C_28_H_23_Cl_3_N_2_O_3_ [M+H]^+^ calculated 541.08470 m/z, found 541.08344 m/z.

### 3.3. Lipophilicity Determination by HPLC

A HPLC separation module Agilent 1200 Series (Agilent Technologies, Santa Clara, CA, USA) equipped with a Dual Absorbance Detector (DAD SL G1315C, Agilent Technologies) was used. A chromatographic column Symmetry^®^ C18 5 μm, 4.6 × 250 mm, Part No. W21751W016 (Waters Corp, Milford, MA, USA) was used. The HPLC separation process was monitored by by ChemStation for LC 3D systems (Agilent Technologies). Isocratic elution by a mixture of MeOH (HPLC grade, 72%) and H_2_O-HPLC Mili-Q grade (28%) as a mobile phase was used for the determination of capacity factor *k*. Isocratic elution by a mixture of MeOH (HPLC grade, 72%) and acetate buffered saline (pH 7.4 and pH 6.5) (28%) as a mobile phase was used for the determination of distribution coefficient expressed as *D*_7.4_ and *D*_6.5_. The total flow of the column was 1.0 mL/min, injection 20 μL, column temperature 40 °C, and sample temperature 10 °C. The detection wavelength of 210 nm was chosen. A KI methanolic solution was used for the determination of the dead times (*t_d_*). Retention times (*t_r_*) were measured in minutes. The capacity factors *k* were calculated according to the formula *k* = (*t_r_* − *t_d_*)/*t_d_*, where *t_R_* is the retention time of the solute, and *t_d_* is the dead time obtained using an unretained analyte. The distribution coefficients *D*_pH_ were calculated according to the formula *D*_pH_ = (*t_r_* − *t_d_*)/*t_d_*. Each experiment was repeated three times. The log *k* values of individual compounds are shown in Table 1.

### 3.4. In Vitro Antibacterial Evaluation

In vitro antibacterial activity of the synthesized compounds was evaluated against representatives of multidrug-resistant bacteria, three clinical isolates of methicillin-resistant *S. aureus*: clinical isolate of animal origin, MRSA 63718 (Department of Infectious Diseases and Microbiology, Faculty of Veterinary Medicine, University of Veterinary Sciences Brno, Czech Republic), carrying the *mecA* gene; and MRSA SA 630 [46] and MRSA SA 3202 [46] (National Institute of Public Health, Prague, Czech Republic), both of human origin. Suspected colonies were confirmed by PCR; a 108bp fragment specific for *S. aureus* was detected [61]. All isolates were tested for the presence of the *mecA* gene encoding methicillin resistance [62]. These three clinical isolates were classified as vancomycin-susceptible (but with higher MIC of vancomycin equal to 2 μg/mL (VA2-MRSA) within the susceptible range for MRSA 63718) methicillin-resistant *S. aureus* (VS-MRSA) [63]. Vancomycin-susceptible methicillin-susceptible *S. aureus* (VS-MSSA) ATCC 29213, obtained from the American Type Culture Collection, was used as the reference and quality control strain. *Enterococcus faecalis* ATCC 29212 was obtained from the American Type Culture Collection and used as the reference and quality control strain.

The minimum inhibitory concentrations (MICs) were evaluated by the microtitration broth method according to the CLSI [64,65] with some modifications. The compounds were dissolved in DMSO (Sigma, St. Louis, MO, USA) to get a concentration 10 µg/mL and diluted in a microtitration plate in an appropriate medium, namely, Cation Adjusted Mueller–Hinton (CaMH, Oxoid, Basingstoke, UK) for staphylococci (*S. aureus* ATCC 29213, clinical isolates of MRSA 63718, SA 630, and SA 3202) and brain heart infusion (BHI, Oxoid) for *E. faecalis* ATCC 29212, to reach the final concentration of 256-0.125 µg/mL. The plate was inoculated by the tested microorganisms. The final concentration of bacterial cells was 10^5^ for bacteria. Ampicillin (Sigma) was used as reference drugs. A drug-free control and a sterility control were included. The plates were incubated for 24 h at 37 °C for staphylococci and *E. faecalis* ATCC 29212 (stability evaluation of the carbamates, see Supplementary materials, Table S1). After static incubation in the darkness in an aerobic atmosphere, the MIC was visually evaluated as the lowest concentration of the tested compound, which completely inhibited the growth of the microorganism. The experiments were repeated three times. The results are summarized in Table 2.

### 3.5. Determination of Minimum Bactericidal Concentrations

For the *S. aureus* and MRSA isolates, the agar aliquot subculture method [66,67] was used as a test for bactericidal agents. After the MIC value determination, the inoculum was transferred to Mueller–Hinton (Oxoid) agar using a multipoint inoculator. The plates were incubated in a thermostat at 37 °C for 24 h. The lowest concentration of test compound at which ≤5 colonies were obtained was then evaluated as MBC, corresponding to a 99.9% decrease in CFU relative to the original inoculum.

### 3.6. MTT Assay

Compounds were prepared as previously stated and diluted in CaMH broth for *S. aureus* to achieve the desired final concentrations. *S. aureus* bacterial suspension in sterile distilled water at 0.5 McFarland was diluted 1:3. Inocula were added to each well by multi-inoculator. Diluted mycobacteria in broth free from inhibiting compounds were used as the growth control. All compounds were prepared in duplicate. Plates were incubated at 37 °C for 24 h for *S. aureus*. After the incubation period, 10% well volume of MTT (3-(4,5-dimethylthiazol-2-yl)-2,5-diphenyltetrazolium bromide) reagent (Sigma) was mixed into each well and incubated at 37 °C 1 h for *S. aureus*. Then 100 µL of 17% sodium dodecyl sulphate in 40% dimethylformamide was added to each well. The plates were read at 570 nm. The absorbance readings from the cells grown in the presence of the tested compounds were compared with uninhibited cell growth to determine the relative percent inhibition. The percent inhibition was determined through the MTT assay. The percent viability is calculated through the comparison of a measured value and that of the uninhibited control: % viability = OD_570E_/OD_570P_ × 100, where OD_570E_ is the reading from the compound-exposed cells, while OD_570P_ is the reading from the uninhibited cells (positive control). Cytotoxic potential is determined by a percent viability of <70% [51,52,68,69]. The results are summarized in Table 3.

### 3.7. Crystal Violet Uptake

The method of crystal violet uptake [53,54] was used to study membrane alteration. A bacterial suspension was cultivated to logarithmic phase in CaMH and harvested at 4500 rpm for 5 min. The cells were washed twice and resuspended in phosphate-buffered saline (PBS) containing 4× MIC of the tested compounds. Tween 20 (1% solution) was used as the positive control. A growth control without antibiotics was included. The tubes were cultivated at 37 °C for 1 h. After that, the tubes were centrifuged at 4500 rpm for 15 min and washed twice in PBS. The cells were resuspended in PBS containing crystal violet (10 µg/mL). After 15 min, the tubes were incubated at 37 °C and centrifuged (15 min, 4500 rpm), and the absorbance of the supernatant at 595 nm was measured. The experiment was repeated five times, and the results were averaged. The percentage of crystal violet uptake was evaluated according to the equation

$$\%\;of\;uptake=\frac{OD_{595}\;of\;sample}{OD_{595}\;of\;crystal\;violet\;solution\;}\times 100$$

### 3.8. In Vitro Cytotoxicity Assay (WST-1 Assay)

The human THP-1 cell line (European Collection of Cell Cultures, Salisbury, UK) was cultured in RPMI 1640 medium supplemented with 10% fetal bovine serum, 2% l-glutamine, 1% penicillin, and streptomycin at 37 °C with 5% CO_2_. Cells were passaged at approximately 1-week intervals [70]. All the tested compounds were prepared and dissolved as mentioned in our previous work [22]. The preliminary in vitro screening of the cytotoxicity of the most effective compounds was performed using the human THP-1 cell line, as described previously [22]. The cytotoxicity was evaluated after 24 h as the IC_50_ value (compound concentration causing 50% inhibition of cell population proliferation), see Table 2.

## 4. Conclusions

Pattern 1-hydroxy-*N*-(2,4,5-trichlorophenyl)-2-naphthamide and the thirteen carbamates derived from it were prepared and characterized. All the compounds were tested against *S. aureus* ATCC 29213 and *E. faecalis* ATCC 29212 as the references and quality control strains. While the compounds did not have anti-enterococcal activity, they showed remarkable antistaphylococcal activity, not only against *S. aureus* but also against MRSA isolates at the nanomolar and submicromolar levels. Although the primary in vitro cytotoxicity performed on the human cancer cell line THP-1 was in the IC_50_ range of 2.61–3.44 μM, the calculated selectivity index for only three compounds was <20. 2-[(2,4,5-Trichlorophenyl)carbamoyl]naphthalen-1-yl ethylcarbamate (**2**) and 2-[(2,4,5-trichlorophenyl)carbamoyl]naphthalen-1-yl butylcarbamate (**4**) showed MICs in the range of 18–64 nM against *S. aureus* and at least two other MRSA isolates, while 2-[(2,4,5-trichlorophenyl)carbamoyl]naphthalen-1-yl heptylcarbamate (**7**) and 2-[(2,4,5-trichlorophenyl)carbamoyl]naphthalen-1-yl (4-phenylbutyl)carbamate (**14**) demonstrated MBCs in the range of 0.124–0.461 μM against all the tested staphylococci. It can be summarized that the MIC and MBC effects against *S. aureus* decrease with increasing lipophilicity and bulkiness of the carbamate tails, while against most of the MRSA isolates the bactericidal activity increases with increasing lipophilicity and length of the carbamate tail, up to C_(7)_, or phenylbutyl chain, and then sharply decreases due to the cut-off effect. The crystal violet uptake assay did not show significant damage to the bacterial wall/membrane. Rather, interactions of the compounds with the bacterial enzyme equipment can be predicted, as demonstrated by the MTT assay. Based on all these observations, it can be concluded that most of the tested carbamates can be considered as promising agents suitable for further study. However, the results obtained in this study will need to be supported by functional genomic and proteomic studies to reveal the mechanism of action of carbamates on staphylococci.

## Figures and Tables

**Table 1:** Structures of 2-[(2,4,5-trichlorophenyl)carbamoyl]naphthalen-1-yl carbamates **2**–**14**, calculated lipophilicities (log *P*/Clog *P*), and experimentally determined log *k*, log *D*_6.5_, and log *D*_7.4_ values of the investigated compounds, as well as their molecular weight (MW), number of H-bond donors (HBD), number of H-bond acceptors (HBA), number of rotatable bonds (RB), and molar volume (MV) of the R tails.

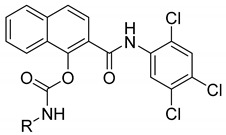												
No.	R	log *P* ^1^	log *P* ^2^	Clog *P* ^2^	log *k*	log *D*_6.5_	log *D*_7.4_	MW ^1^	HBD ^1^	HBA ^1^	RB ^1^	MV ^1^ [cm^3^]
**1**	–	6.31	5.12	5.9945	0.6600	0.5782	0.5384	366.62	2	3	2	–
**2**	Et	5.63	5.34	5.1356	0.8249	0.8294	0.8339	437.70	2	5	5	76.10
**3**	Pr	5.80	5.83	5.6646	0.9689	0.9734	0.9793	451.73	2	5	6	92.61
**4**	Bu	6.15	6.25	6.1936	1.0756	0.9980	1.1401	465.76	2	5	7	109.11
**5**	Pen	7.45	6.66	6.7226	1.3051	1.3072	1.3168	479.78	2	5	8	125.62
**6**	Hex	7.83	7.08	7.2516	1.4883	1.4899	1.4990	493.81	2	5	9	142.13
**7**	Hep	8.46	7.50	7.7806	1.6741	1.6765	1.6863	507.84	2	5	10	158.63
**8**	Oct	8.84	7.92	8.3096	1.8654	1.8687	1.8785	521.86	2	5	11	175.14
**9**	iPr	5.73	5.66	5.4446	0.9526	0.9579	1.0468	451.73	2	5	5	92.98
**10**	cPent	6.40	6.14	6.0786	1.1481	1.1521	1.1515	477.77	2	5	5	112.65
**11**	cHex	6.71	6.55	6.6376	1.2911	1.2952	1.2955	491.79	2	5	5	129.17
**12**	cHep	6.77	6.97	7.1966	1.4469	1.4492	1.4527	505.82	2	5	5	145.64
**13**	PhEt	6.69	7.02	6.7036	1.2450	1.2473	1.2470	513.80	2	5	7	136.80
**14**	PhBu	7.66	7.85	7.6116	1.5167	1.5193	1.5191	541.85	2	5	9	169.81
**Ro5**		**<5**	**<5**	**–**	**–**	**–**	**–**	**<500**	**<5**	**<10**	**–**	**–**

^1^ ACD/Percepta ver. 2012 (Advanced Chemistry Development. Inc., Toronto, ON, Canada, 2012); ^2^ ChemBioDrawUltra 13.0 (CambridgeSoft, PerkinElmer Inc., Waltham, MA, USA); Ro5 = Lipinski’s Rule of Five.

**Table 2:** In vitro antistaphylococcal and anti-enterococcal activities (MIC/MBC (μM)) compared to ampicillin (AMP) and ciprofloxacin (CPX), and the in vitro cell viability assay (IC_50_ (µM) ± SD, *n* = 6) on the human monocytic leukemia cell line (THP-1) compared to camptothecin (CMT).

No.	µM										SI (IC_50_/ MIC *)
	SA		MRSA1		MRSA2		MRSA3		EF	THP-1	
	MIC	MBC	MIC	MBC	MIC	MBC	MIC	MBC	MIC	IC_50_	
**1**	698	–	698	–	698	–	698	–	477	>30	–
**2**	0.018 *	**0.018**	0.144	**0.286**	0.018	0.144	0.018	**0.073**	36.6	2.85 ± 0.17	156
**3**	0.035 *	**0.035**	0.139	**0.277**	0.553	**0.553**	0.071	**0.071**	283	3.12 ± 0.07	88.1
**4**	0.034 *	**0.034**	0.135	**0.268**	0.017	0.537	0.069	**0.069**	275	3.42 ± 0.10	99.6
**5**	0.067 *	**0.067**	0.261	**0.261**	0.261	**0.521**	0.131	**0.131**	267	3.44 ± 0.04	51.3
**6**	0.128 *	**0.128**	0.253	**0.253**	0.253	**0.506**	0.253	**0.253**	259	3.20 ± 0.19	25.1
**7**	0.124 *	**0.124**	0.246	**0.246**	0.246	**0.492**	0.246	**0.246**	63.0	3.19 ± 0.44	25.7
**8**	0.479 *	**0.958**	0.958	**1.916**	0.479	**1.916**	1.921	**1.921**	123	2.61 ± 0.32	5.5
**9**	0.018 *	**0.018**	0.139	**0.139**	0.035	0.277	0.277	**0.277**	142	3.26 ± 0.08	184
**10**	0.262 *	**0.262**	0.262	**0.262**	0.262	**1.047**	2.090	**2.090**	268	3.23 ± 0.29	12.3
**11**	0.128 *	**0.508**	0.254	**0.508**	0.508	**1.017**	0.508	**0.508**	65.1	2.76 ± 0.24	21.5
**12**	0.494 *	**0.494**	0.247	**0.247**	0.494	**0.494**	0.494	**0.494**	127	3.24 ± 0.12	6.6
**13**	0.062 *	**0.062**	0.123	**0.123**	0.062	0.973	0.123	**0.243**	125	2.79 ± 0.28	44.8
**14**	0.116 *	**0.116**	0.231	**0.231**	0.116	**0.461**	0.231	**0.461**	118	2.73 ± 0.37	23.5
**APM**	5.72	>5.72	45.8	>45.8	45.8	>45.8	45.8	>45.8	11.5	–	–
**CPX**	0.75	0.75	12.1	12.1	386	386	28.1	24.1	1.51	–	–
**CMT**	–	–	–	–	–	–	–	–	–	0.16 ± 0.07	–

SA *= Staphylococcus aureus* ATCC 29213; MRSA1–3 *=* clinical isolates of methicillin-resistant *S. aureus* 63718 (Department of Infectious Diseases and Microbiology, Faculty of Veterinary Medicine, University of Veterinary Sciences Brno, Czech Republic), and SA 630, SA 3202 (National Institute of Public Health, Prague, Czech Republic); EF = *Enterococcus faecalis* ATCC 29213; * = MIC values used for calculation of selectivity index (SI). The real bactericidal values required by the MBC/MIC ≤ 4 rule are in bold.

**Table 3:** Lowest MIC values with at least 70% inhibition of *S. aureus* ATCC 29213 viability.

No.	Conc.	*S. aureus* Viability Inhibition (%)
**2**	4× MIC (4× MBC)	82.3
**3**	4× MIC (4× MBC)	94.3
**4**	8× MIC (8× MBC)	92.9
**7**	4× MIC (4× MBC)	95.2
**8**	2× MIC (1× MBC)	75.0
**9**	2× MIC (2× MBC)	95.7
**13**	4× MIC (4× MBC)	95.7
**14**	2× MIC (2× MBC)	95.7
**APM**	8× MIC (>8× MBC)	90.0
**CPX**	32× MIC (32× MBC)	92.8

## Data Availability

Data is contained within the article and supplementary material.

## Supplementary Materials

The following supporting information can be downloaded at: https://www.mdpi.com/article/10.3390/ph15060715/s1, Figure S1: Mutual comparison of experimentally determined values of log *k*, log *D*_6.5_ and log *D*_7.4_ of all prepared compounds **1**–**14**; Figure S2: Comparison of experimentally determined values of log *k*, log *D*_6.5_ and log *D*_7.4_ of prepared compounds with predicted Clog *P* (ChemDrawUltra) values; Figure S3: Comparison of experimentally determined values of log *k*, log *D*_6.5_ and log *D*_7.4_ of prepared compounds with predicted log *P* (ACD/Percepta) values; Figure S4: Comparison of experimentally determined values of log *k*, log *D*_6.5_ and log *D*_7.4_ of prepared compounds with predicted log *P* (ChemDrawUltra) values; Figure S5: ^1^H-NMR (DMSO-*d_6_*) spectrum of 2-[*N*-(2,4,5-trichlorophenyl)carbamoyl]naphthalen-1-yl ethyl carbamate (**2**); Figure S6: ^13^C-NMR (DMSO-*d_6_*) spectrum of 2-[*N*-(2,4,5-trichlorophenyl)carbamoyl]naphthalen-1-yl ethyl carbamate (**2**); Figure S7: ^1^H-NMR (DMSO-*d_6_*) spectrum of 2-[*N*-(2,4,5-trichlorophenyl)carbamoyl]naphtha- len-1-yl propyl carbamate (**3**); Figure S8: ^13^C-NMR (DMSO-*d_6_*) spectrum of 2-[*N*-(2,4,5-trichlorophenyl)carbamoyl]naphthalen-1-yl propyl carbamate (**3**); Figure S9: ^1^H-NMR (DMSO-*d_6_*) spectrum of 2-[*N*-(2,4,5-trichlorophenyl)carbamoyl]naphthalen-1-yl butyl carbamate (**4**); Figure S10: ^13^C-NMR (DMSO-*d_6_*) spectrum of 2-[*N*-(2,4,5-trichlorophenyl)carbamoyl]naphtha- len-1-yl butyl carbamate (**4**); Figure S11: ^1^H-NMR (DMSO-*d_6_*) spectrum of 2-[*N*-(2,4,5-trichlorophenyl)carbamoyl]naphthalen-1-yl pentyl carbamate (**5**); Figure S12: ^13^C-NMR (DMSO-*d_6_*) spectrum of 2-[*N*-(2,4,5-trichlorophenyl)carbamoyl]naphthalen-1-yl pentyl carbamate (**5**); Figure S13: ^1^H-NMR (DMSO-*d_6_*) spectrum of 2-[*N*-(2,4,5-trichlorophenyl)carbamoyl]naphtha- len-1-yl hexyl carbamate (**6**); Figure S14: ^13^C-NMR (DMSO-*d_6_*) spectrum of 2-[*N*-(2,4,5-trichlorophenyl)carbamoyl]naphtha- len-1-yl hexyl carbamate (**6**); Figure S15: ^1^H-NMR (DMSO-*d_6_*) spectrum of 2-[*N*-(2,4,5-trichlorophenyl)carbamoyl]naphthalen-1-yl heptyl carbamate (**7**); Figure S16: ^13^C-NMR (DMSO-*d_6_*) spectrum of 2-[*N*-(2,4,5-trichlorophenyl)carbamoyl]naphtha- len-1-yl heptyl carbamate (**7**); Figure S17: ^1^H-NMR (DMSO-*d_6_*) spectrum of 2-[*N*-(2,4,5-trichlorophenyl)carbamoyl]naphtha- len-1-yl octyl carbamate (**8**); Figure S18: ^13^C-NMR (DMSO-*d_6_*) spectrum of 2-[*N*-(2,4,5-trichlorophenyl)carbamoyl]naphthalen-1-yl octyl carbamate (**8**); Figure S19: ^1^H-NMR (DMSO-*d_6_*) spectrum of 2-[*N*-(2,4,5-trichlorophenyl)carbamoyl]naphtha- len-1-yl isopropyl carbamate (**9**); Figure S20: ^13^C-NMR (DMSO-*d_6_*) spectrum of 2-[*N*-(2,4,5-trichlorophenyl)carbamoyl]naphthalen-1-yl isopropyl carbamate (**9**); Figure S21: ^1^H-NMR (DMSO-*d_6_*) spectrum of 2-[*N*-(2,4,5-trichlorophenyl)carbamoyl]naphthalen-1-yl cyclopentyl carbamate (**10**); Figure S22: ^13^C-NMR (DMSO-*d_6_*) spectrum of 2-[*N*-(2,4,5-trichlorophenyl)- carbamoyl]naphthalen-1-yl cyclopentyl carbamate (**10**); Figure S23: ^1^H-NMR (DMSO-*d_6_*) spectrum of 2-[*N*-(2,4,5-trichlorophenyl)carbamoyl]naphthalen-1-yl cyclohexyl carbamate (**11**); Figure S24: ^13^C-NMR (DMSO-*d_6_*) spectrum of 2-[*N*-(2,4,5-trichlorophenyl)carbamoyl]naphthalen-1-yl cyclohexyl carbamate (**11**); Figure S25: ^1^H-NMR (DMSO-*d_6_*) spectrum of 2-[*N*-(2,4,5-trichlorophenyl)- carbamoyl]naphthalen-1-yl cycloheptyl carbamate (**12**); Figure S26: ^13^C-NMR (DMSO-*d_6_*) spectrum of 2-[*N*-(2,4,5-trichlorophenyl)carbamoyl]naphthalen-1-yl cycloheptyl carbamate (**12**); Figure S27: ^1^H-NMR (DMSO-*d_6_*) spectrum of 2-[*N*-(2,4,5-trichlorophenyl)carbamoyl]naphthalen-1-yl 2-phenylethyl carbamate (**13**); Figure S28: ^13^C-NMR (DMSO-*d_6_*) spectrum of 2-[*N*-(2,4,5-trichloro- phenyl)carbamoyl]naphthalen-1-yl 2-phnylethyl carbamate (**13**); Figure S29: ^1^H-NMR (DMSO-*d_6_*) spectrum of 2-[*N*-(2,4,5-trichlorophenyl)carbamoyl]naphthalen-1-yl 4-phenylbutyl carbamate (**14**); Figure S30: ^13^C-NMR (DMSO-*d_6_*) spectrum of 2-[*N*-(2,4,5-trichlorophenyl)carbamoyl]naphtha- len-1-yl 2-phnylbutyl carbamate (**14**); Table S1: Ethyl carbamate **2** decomposition rate and half-life values.
