# Supplementary material for: Antistaphylococcal Activities and ADME-Related Properties of Chlorinated Arylcarbamoylnaphthalenylcarbamates
Source: Pharmaceuticals (Basel). 2022 Jun 5;15(6):715. doi: 10.3390/ph15060715 (PMC9228535; doi:10.3390/ph15060715)
Supplement: Supplementary file 1 [file pharmaceuticals-15-00715-s001.zip › pharmaceuticals-1738032-supplementary.pdf]

Supplementary Materials

Anti-staphylococcal Activities and ADME-Related Properties of Chlorinated Arylcarbamoylnaphthalenyl-carbamates

Tomas Gonec [1,*], Dominika Pindjakova [2], Lucia Vrablova [2], Tomas Strharsky [1], Hana Michnova [3], Tereza Kauerova [4], Peter Kollar [4,*], Michal Oravec [5], Izabela Jendrzejewska [6], Alois Cizek [3] and Josef Jampilek [2,7]

[1] Department of Chemical Drugs, Faculty of Pharmacy, Masaryk University, Palackeho 1946/1, 612 00 Brno, Czech Republic, strharsky.t@gmail.com (T.S.)

[2] Department of Analytical Chemistry, Faculty of Natural Sciences, Comenius University, Ilkovicova 6, 842 15 Bratislava, Slovakia; pindjakova.dominika@gmail.com (D.P.); lucia.vrablova26@gmail.com (L.V.); josef.jampilek@gmail.com (J.J.)

[3] Department of Infectious Diseases and Microbiology, Faculty of Veterinary Medicine, University of Veterinary Sciences Brno, Palackeho 1946/1, 612 42 Brno, Czech Republic; michnova.hana@gmail.com (H.M.); cizeka@vfu.cz (A.C.)

[4] Department of Pharmacology and Toxicology, Faculty of Pharmacy, Masaryk University, Palackeho 1946/1, 612 00 Brno, Czech Republic; kauerovat@pharm.muni.cz (T.K.)

[5] Global Change Research Institute CAS, Belidla 986/4a, 60300 Brno, Czech Republic; oravec.m@czechglobe.cz

[6] Institute of Chemistry, University of Silesia, Bankowa 12, 40 007 Katowice, Poland; izabela.jendrzejewska@us.edu.pl

[7] Department of Chemical Biology, Faculty of Science, Palacky University Olomouc, Slechtitelu 27, 783 71 Olomouc, Czech Republic

* Correspondence: t.gonec@seznam.cz (T.G.); kollarp@pharm.muni.cz (P.K.)

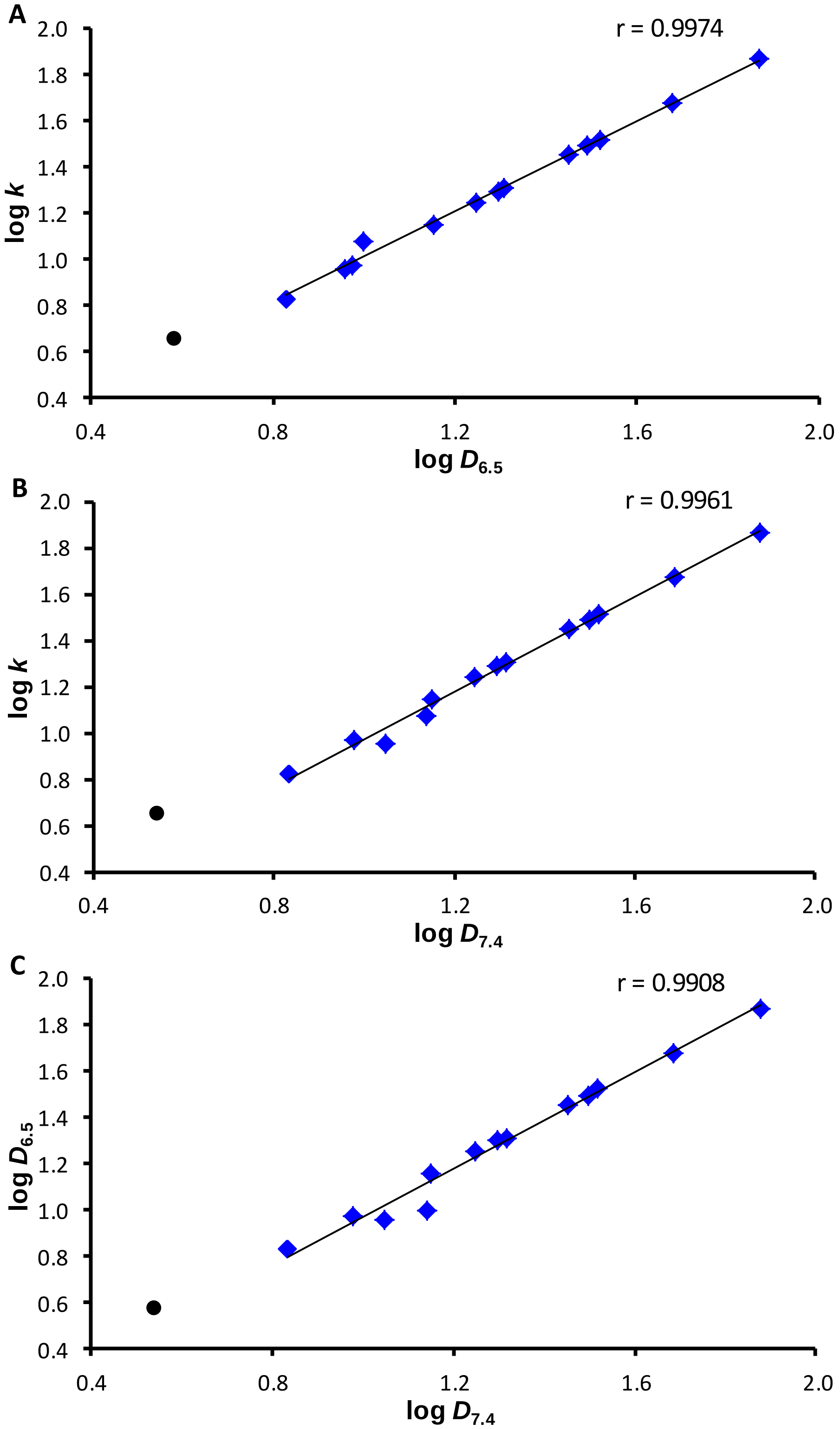

Figure S1. Mutual comparison of experimentally determined values of log *k*, log $D_{6.5}$ and log $D_{7.4}$ of all prepared compounds **1**–**14**. (black circle = anilide **1**, blue rhombuses = carbamates **2**–**14**)

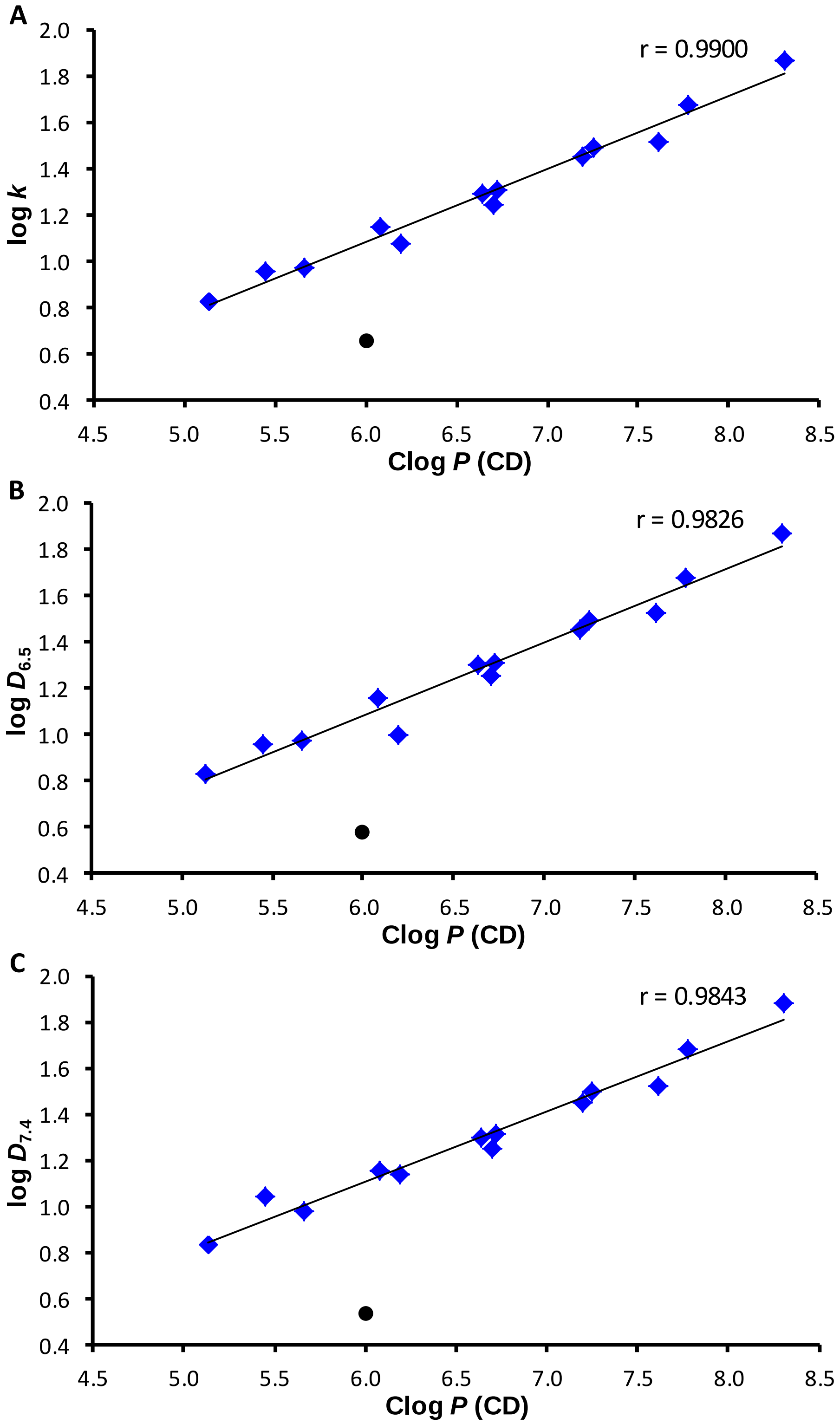

Figure S2. Comparison of experimentally determined values of log *k*, log $D_{6.5}$ and log $D_{7.4}$ of prepared compounds with predicted Clog *P* (ChemDrawUltra) values. (black circle = anilide **1**, blue rhombuses = carbamates **2–14**)

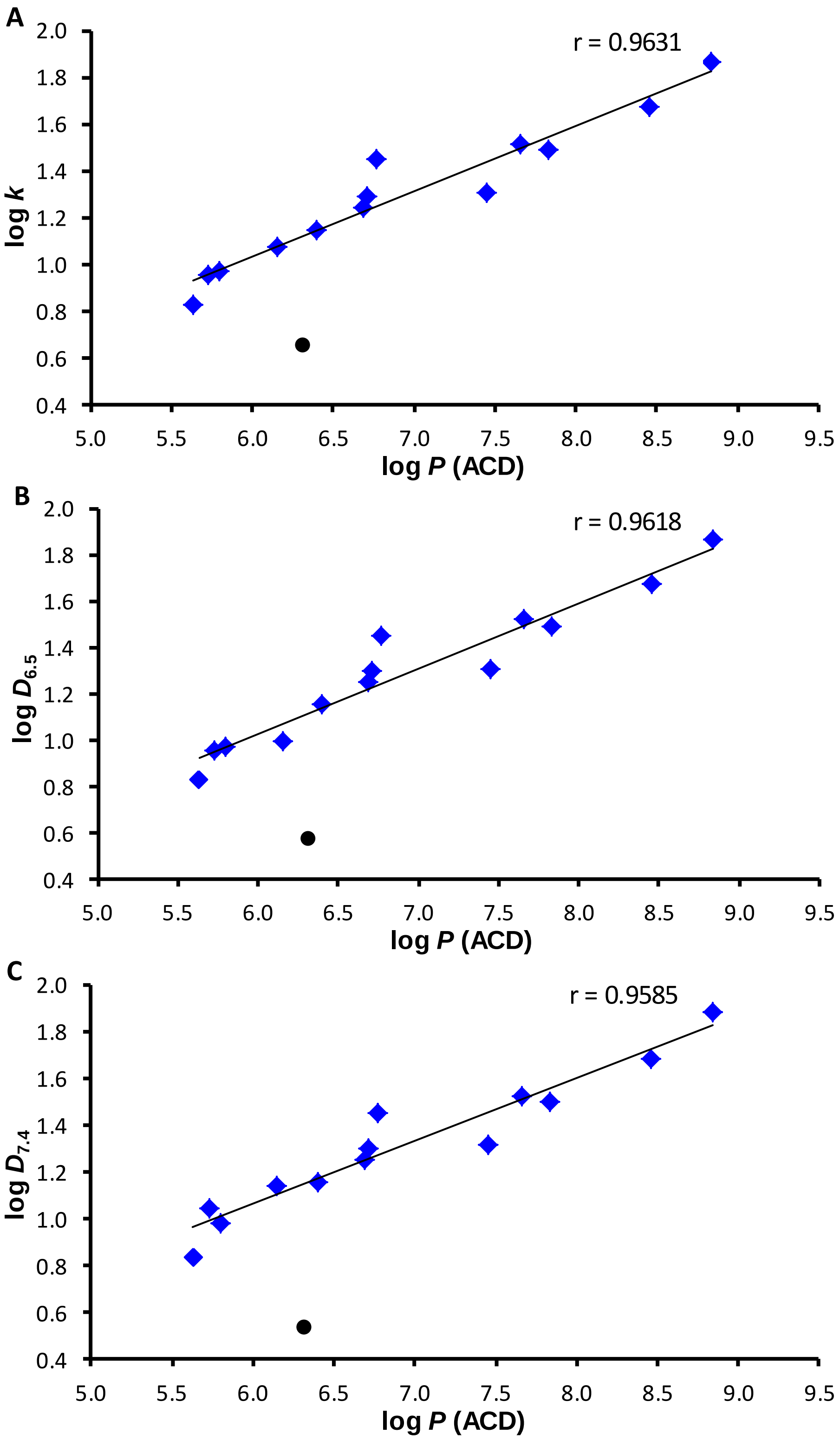

Figure S3. Comparison of experimentally determined values of log *k*, log $D_{6.5}$ and log $D_{7.4}$ of prepared compounds with predicted log *P* (ACD/Percepta) values. (black circle = anilide **1**, blue rhombuses = carbamates **2–14**)

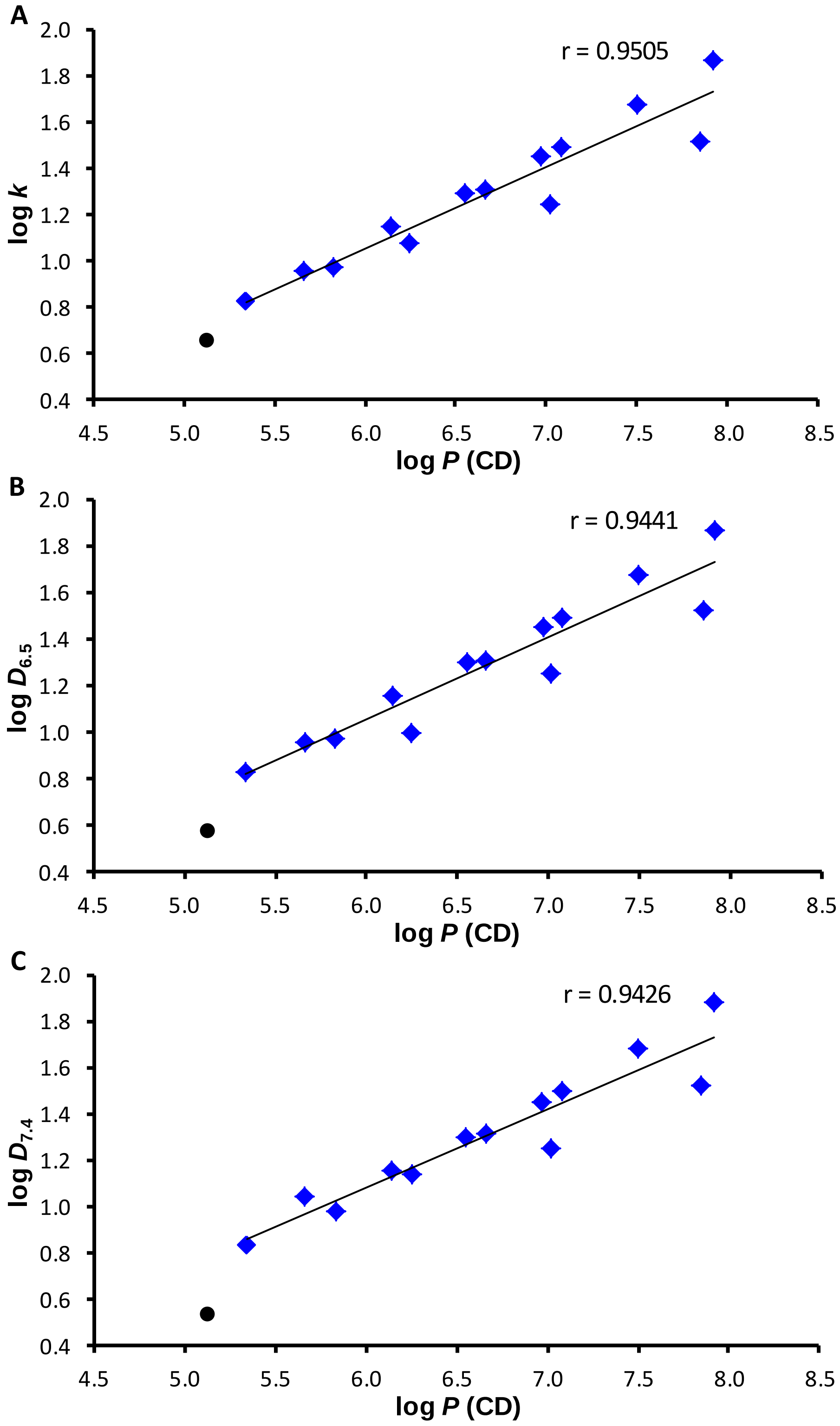

Figure S4. Comparison of experimentally determined values of log *k*, log $D_{6.5}$ and log $D_{7.4}$ of prepared compounds with predicted log *P* (ChemDrawUltra) values. (black circle = anilide **1**, blue rhombuses = carbamates **2–14**)

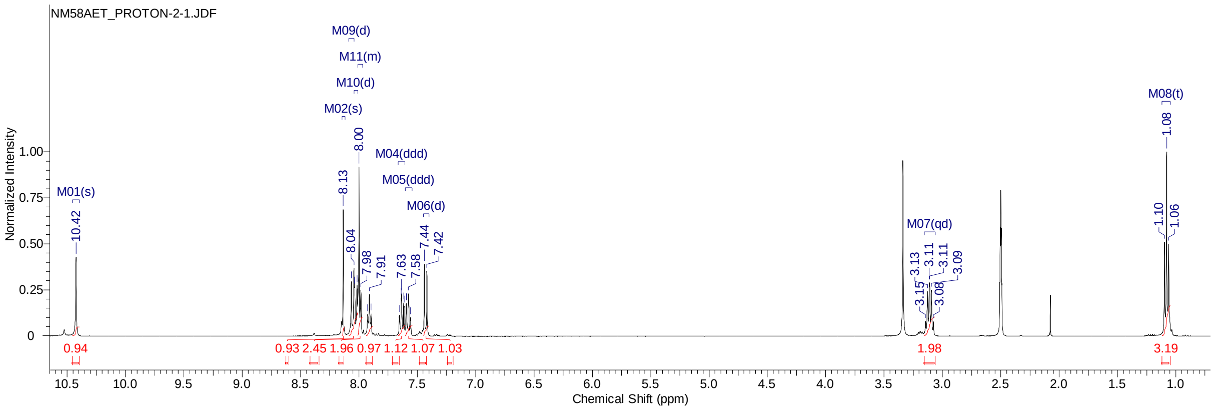

Figure S5. ^{1}H-NMR (DMSO-*d*$_6$) spectrum of 2-[*N*-(2,4,5-trichlorophenyl)carbamoyl]naphthalen-1-yl ethyl carbamate (**2**).

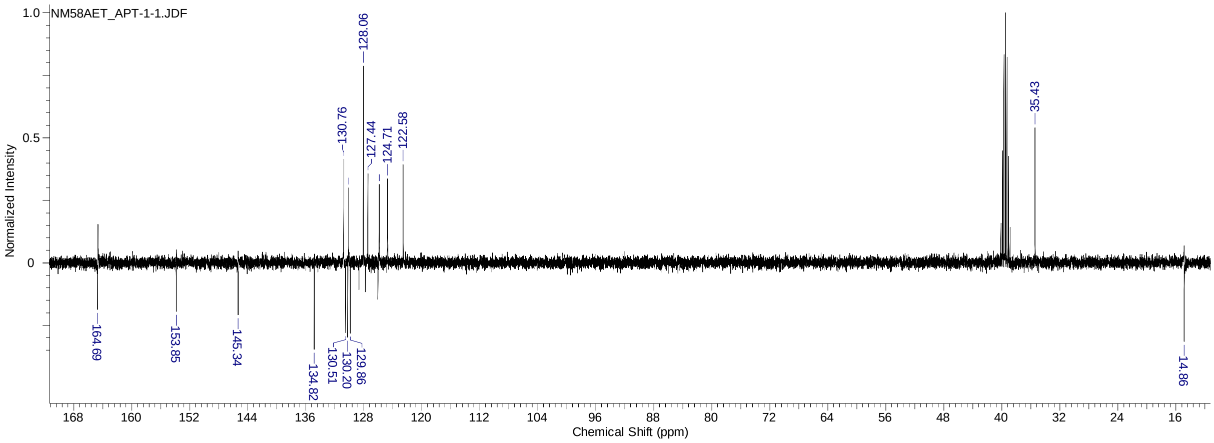

Figure S6. ^{13}C-NMR (DMSO-*d*$_6$) spectrum of 2-[*N*-(2,4,5-trichlorophenyl)carbamoyl]naphthalen-1-yl ethyl carbamate (**2**).

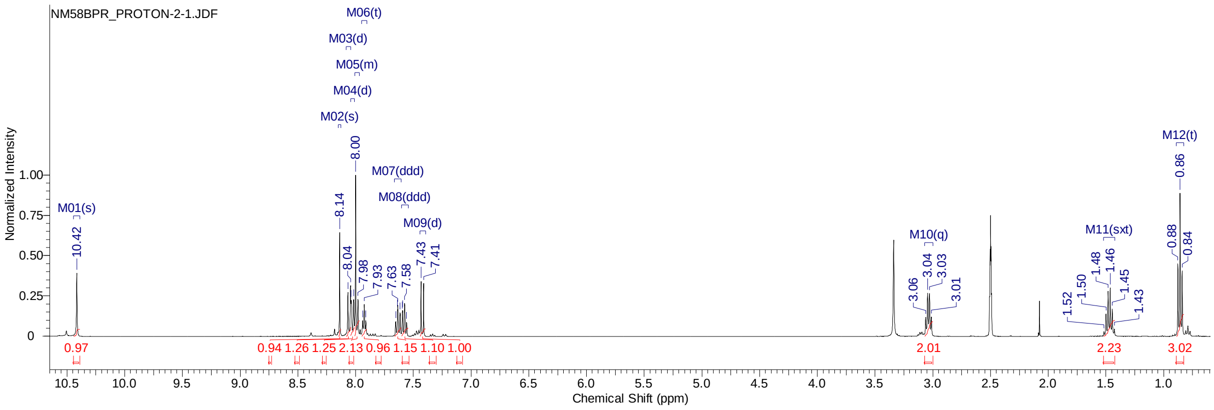

Figure S7. ^{1}H-NMR (DMSO-*d*$_6$) spectrum of 2-[*N*-(2,4,5-trichlorophenyl)carbamoyl]naphthalen-1-yl propyl carbamate (**3**).

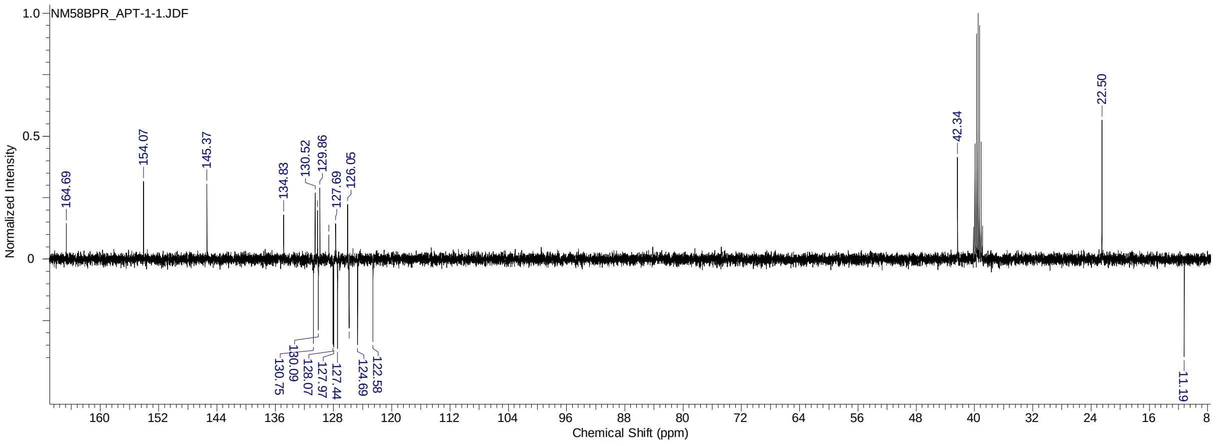

Figure S8. ^{13}C-NMR (DMSO-d_6) spectrum of 2-[*N*-(2,4,5-trichlorophenyl)carbamoyl]naphthalen-1-yl propyl carbamate (**3**).

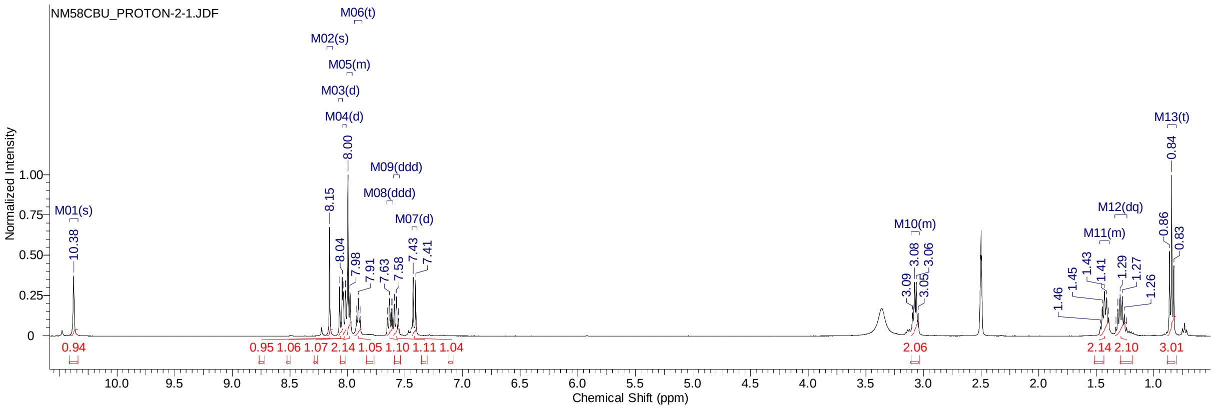

Figure S9. ^{1}H-NMR (DMSO-d_6) spectrum of 2-[*N*-(2,4,5-trichlorophenyl)carbamoyl]naphthalen-1-yl butyl carbamate (**4**).

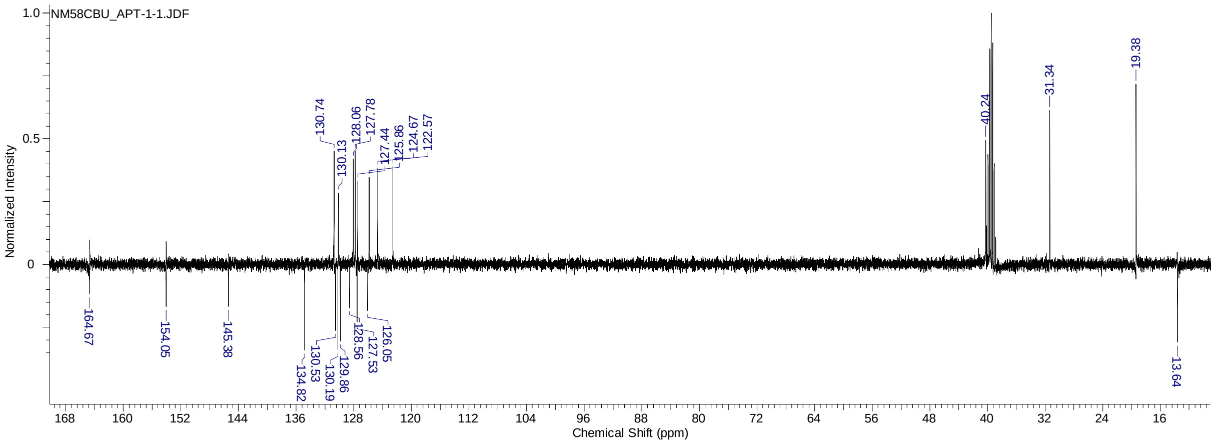

Figure S10. ^{13}C-NMR (DMSO-d_6) spectrum of 2-[*N*-(2,4,5-trichlorophenyl)carbamoyl]naphthalen-1-yl butyl carbamate (**4**).

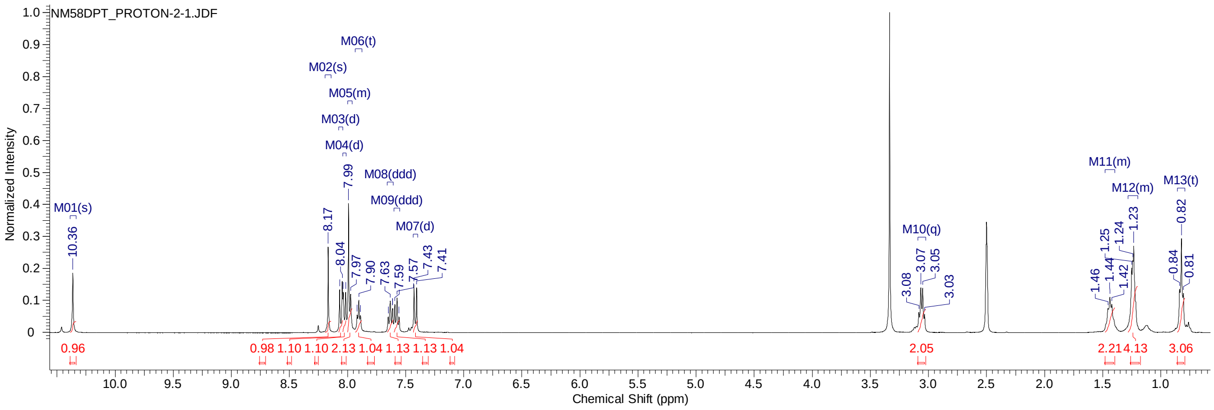

Figure S11. ^{1}H-NMR (DMSO-d_6) spectrum of 2-[*N*-(2,4,5-trichlorophenyl)carbamoyl]naphthalen-1-yl pentyl carbamate (**5**).

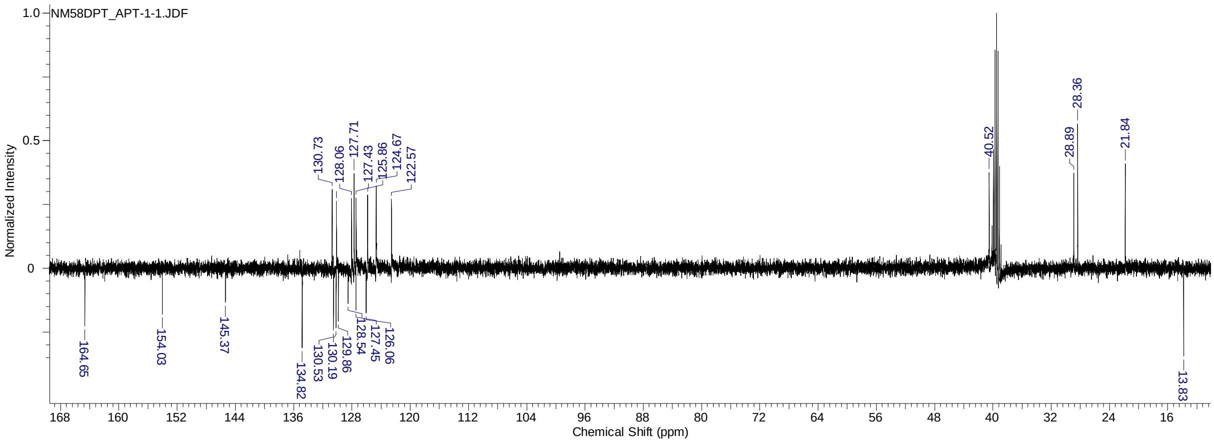

Figure S12. ^{13}C-NMR (DMSO-d_6) spectrum of 2-[*N*-(2,4,5-trichlorophenyl)carbamoyl]naphthalen-1-yl pentyl carbamate (**5**).

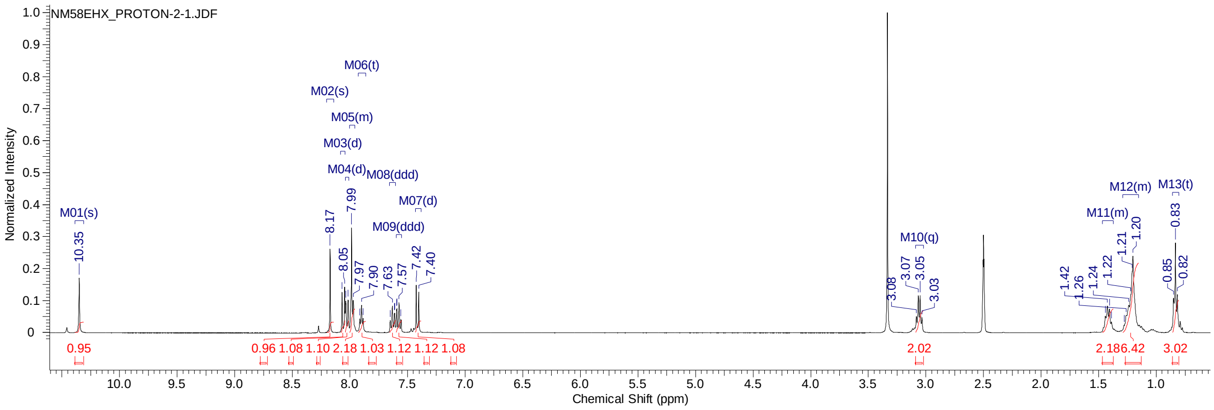

Figure S13. ^{1}H-NMR (DMSO-d_6) spectrum of 2-[*N*-(2,4,5-trichlorophenyl)carbamoyl]naphthalen-1-yl hexyl carbamate (**6**).

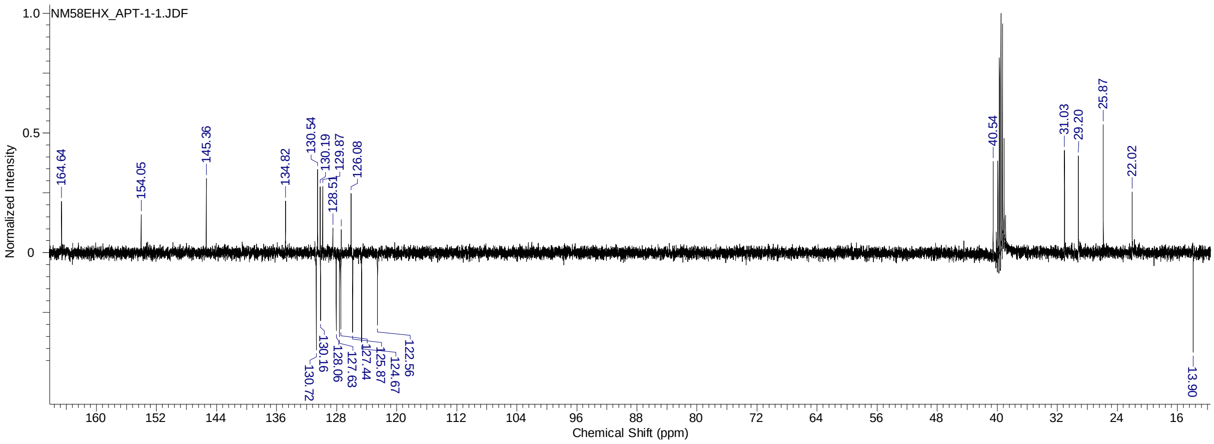

Figure S14. ^{13}C-NMR (DMSO-d_6) spectrum of 2-[*N*-(2,4,5-trichlorophenyl)carbamoyl]naphthalen-1-yl hexyl carbamate (**6**).

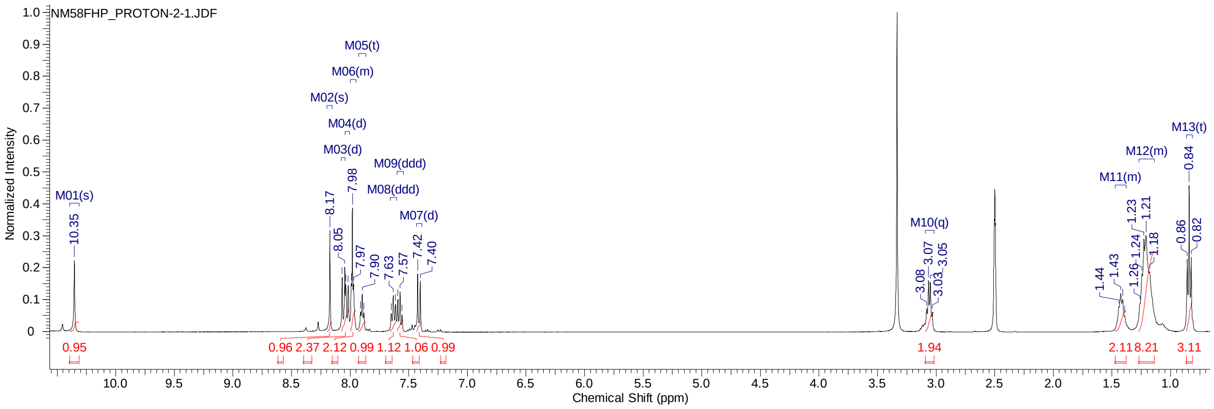

Figure S15. ^{1}H-NMR (DMSO-d_6) spectrum of 2-[*N*-(2,4,5-trichlorophenyl)carbamoyl]naphthalen-1-yl heptyl carbamate (**7**).

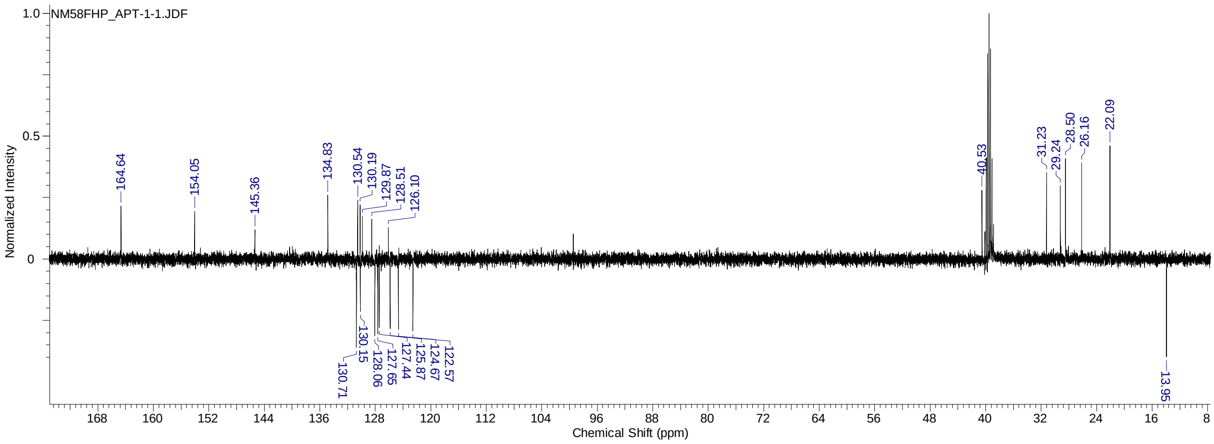

Figure S16. ^{13}C-NMR (DMSO-d_6) spectrum of 2-[*N*-(2,4,5-trichlorophenyl)carbamoyl]naphthalen-1-yl heptyl carbamate (**7**).

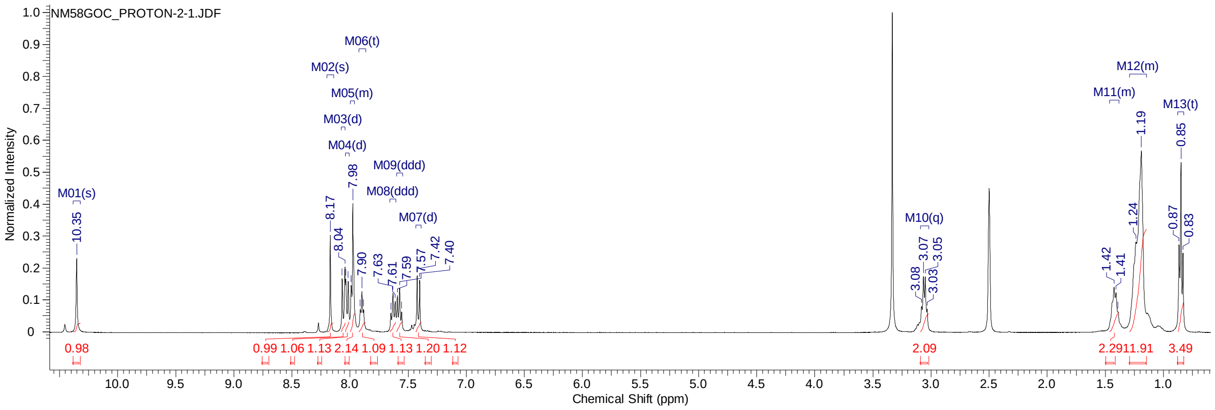

Figure S17. ^{1}H-NMR (DMSO-d_6) spectrum of 2-[*N*-(2,4,5-trichlorophenyl)carbamoyl]naphthalen-1-yl octyl carbamate (**8**).

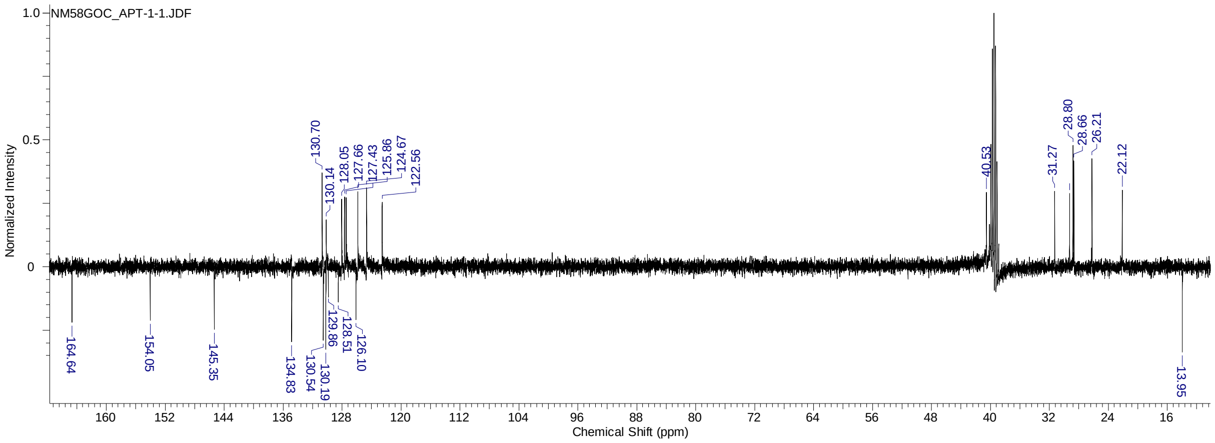

Figure S18. ^{13}C-NMR (DMSO-d_6) spectrum of 2-[*N*-(2,4,5-trichlorophenyl)carbamoyl]naphthalen-1-yl octyl carbamate (**8**).

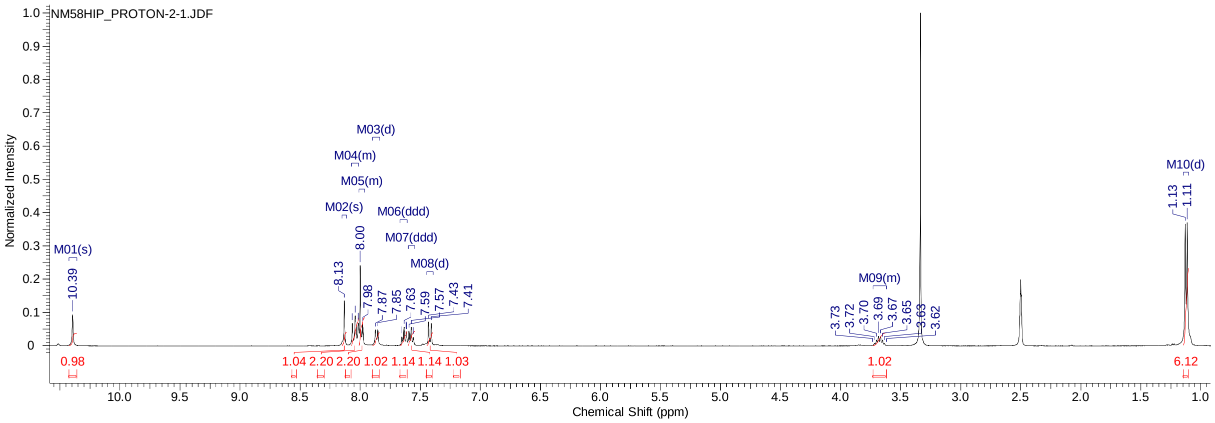

Figure S19. ^{1}H-NMR (DMSO-d_6) spectrum of 2-[*N*-(2,4,5-trichlorophenyl)carbamoyl]naphthalen-1-yl isopropyl carbamate (**9**).

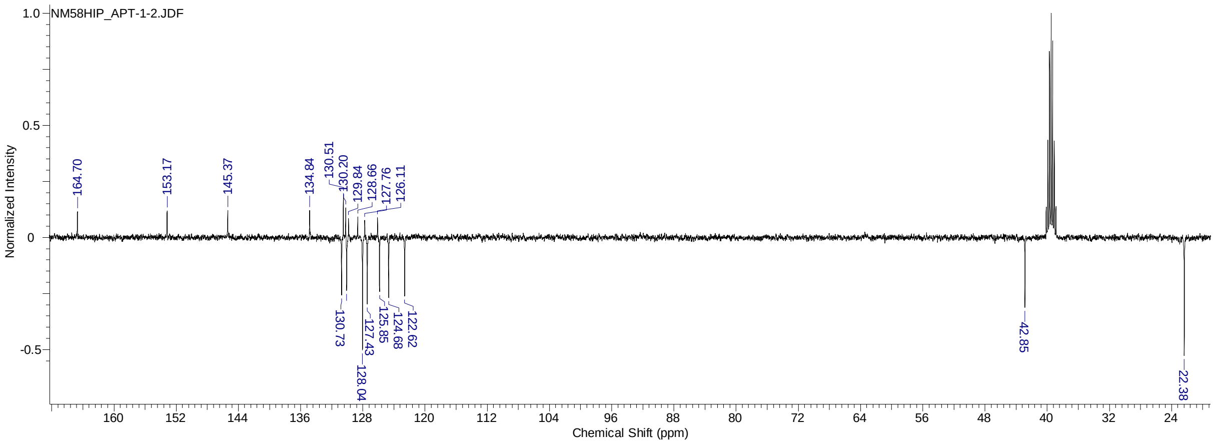

Figure S20. ^{13}C-NMR (DMSO-d_6) spectrum of 2-[*N*-(2,4,5-trichlorophenyl)carbamoyl]naphthalen-1-yl isopropyl carbamate (**9**).

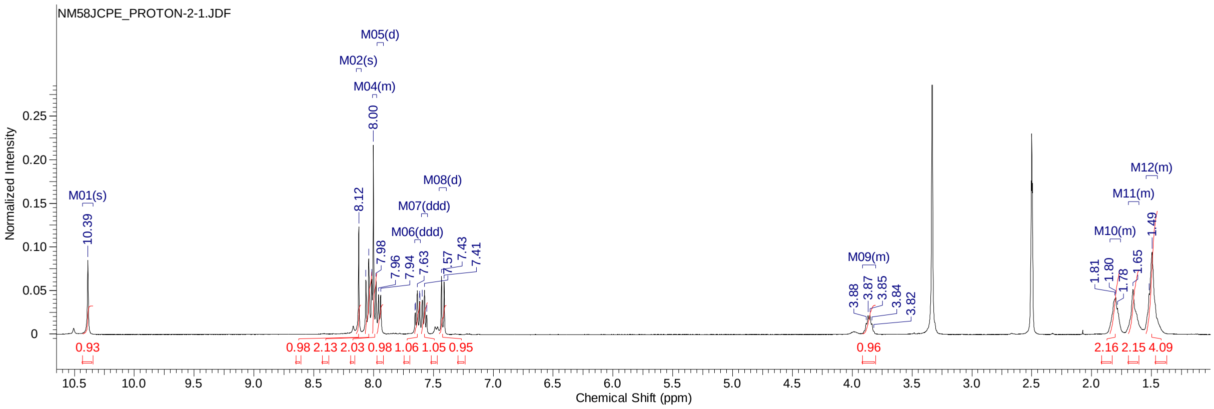

Figure S21. ^{1}H-NMR (DMSO-d_6) spectrum of 2-[*N*-(2,4,5-trichlorophenyl)carbamoyl]naphthalen-1-yl cyclopentyl carbamate (**10**).

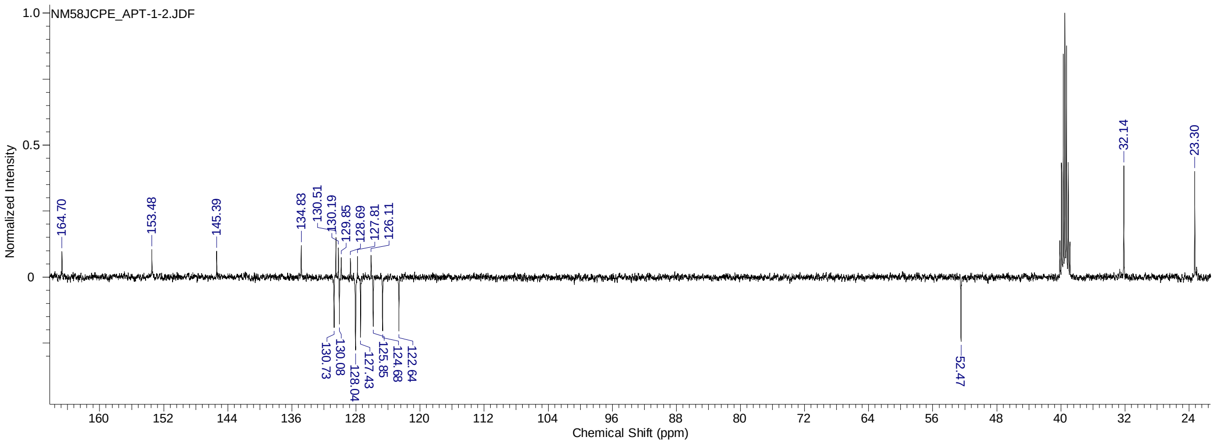

Figure S22. ^{13}C-NMR (DMSO-d_6) spectrum of 2-[*N*-(2,4,5-trichlorophenyl)carbamoyl]naphthalen-1-yl cyclopentyl carbamate (**10**).

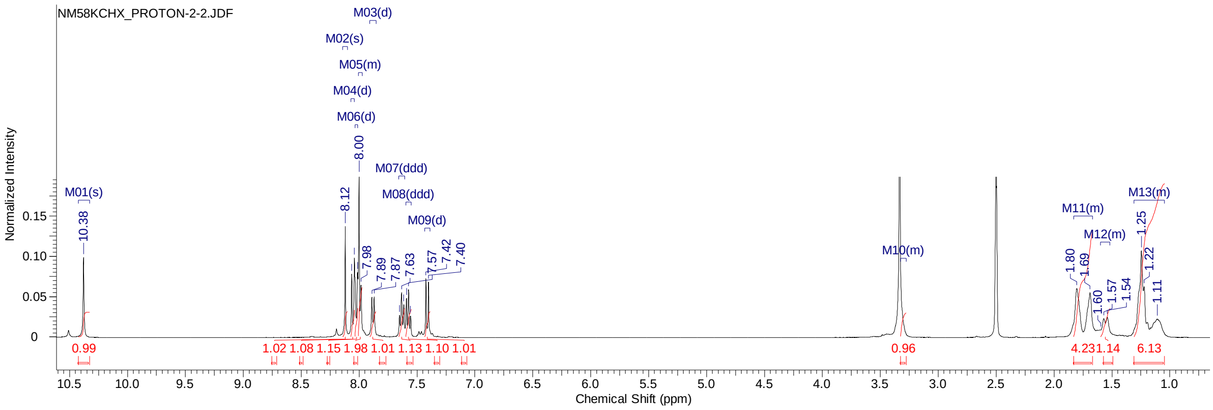

Figure S23. ^{1}H-NMR (DMSO-d_6) spectrum of 2-[*N*-(2,4,5-trichlorophenyl)carbamoyl]naphthalen-1-yl cyclohexyl carbamate (**11**).

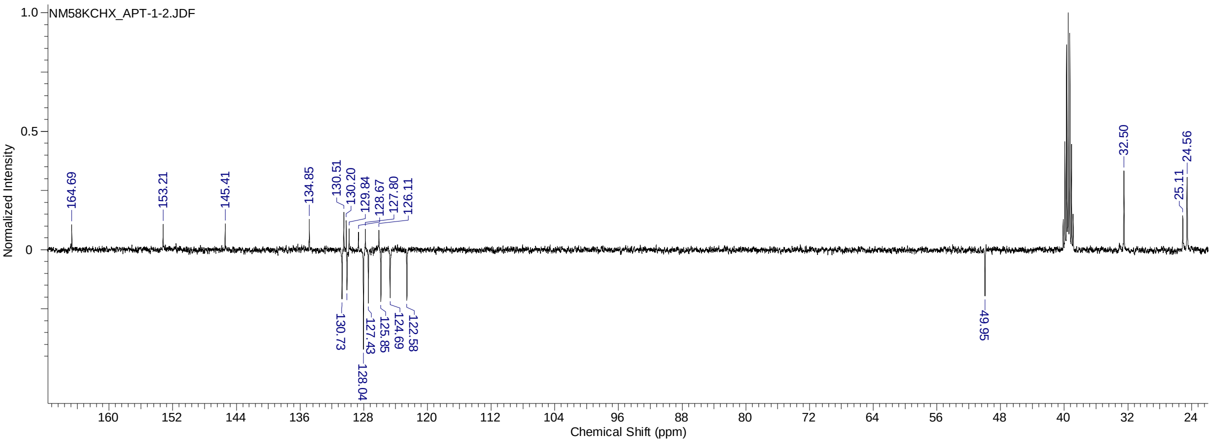

Figure S24. ^{13}C-NMR (DMSO-d_6) spectrum of 2-[*N*-(2,4,5-trichlorophenyl)carbamoyl]naphthalen-1-yl cyclohexyl carbamate (**11**).

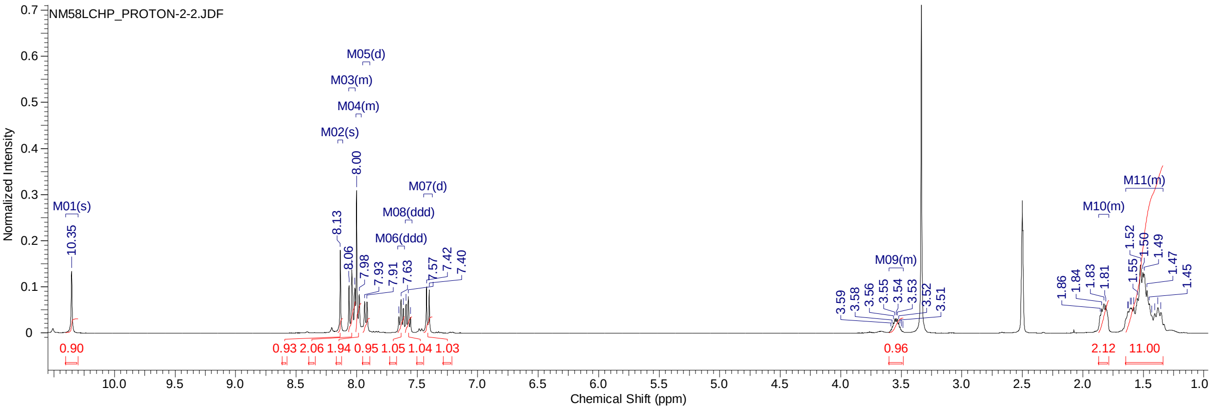

Figure S25. ^{1}H-NMR (DMSO-d_6) spectrum of 2-[*N*-(2,4,5-trichlorophenyl)carbamoyl]naphthalen-1-yl cycloheptyl carbamate (**12**).

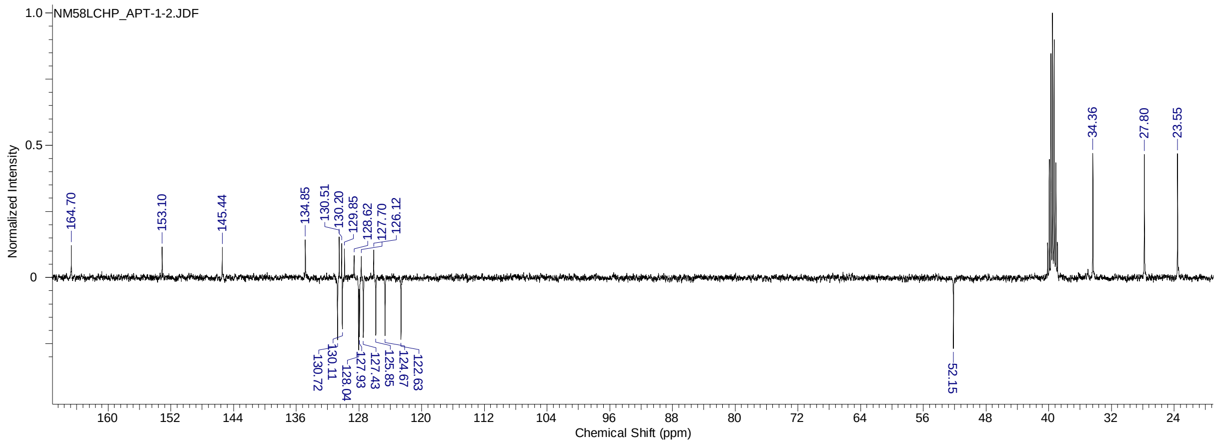

Figure S26. ^{13}C-NMR (DMSO-d_6) spectrum of 2-[*N*-(2,4,5-trichlorophenyl)carbamoyl]naphthalen-1-yl cycloheptyl carbamate (**12**).

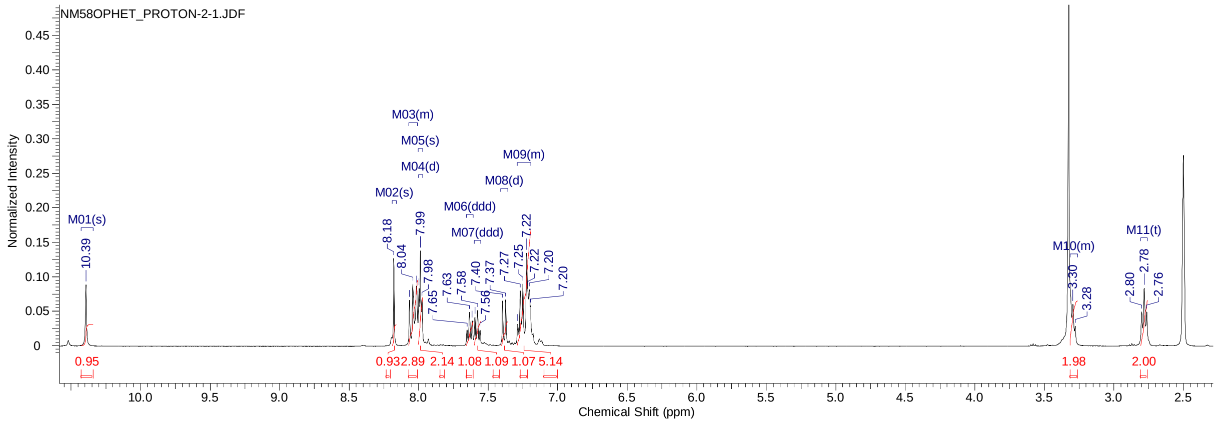

Figure S27. ^{1}H-NMR (DMSO-d_6) spectrum of 2-[*N*-(2,4,5-trichlorophenyl)carbamoyl]naphthalen-1-yl 2-phenylethyl carbamate (**13**).

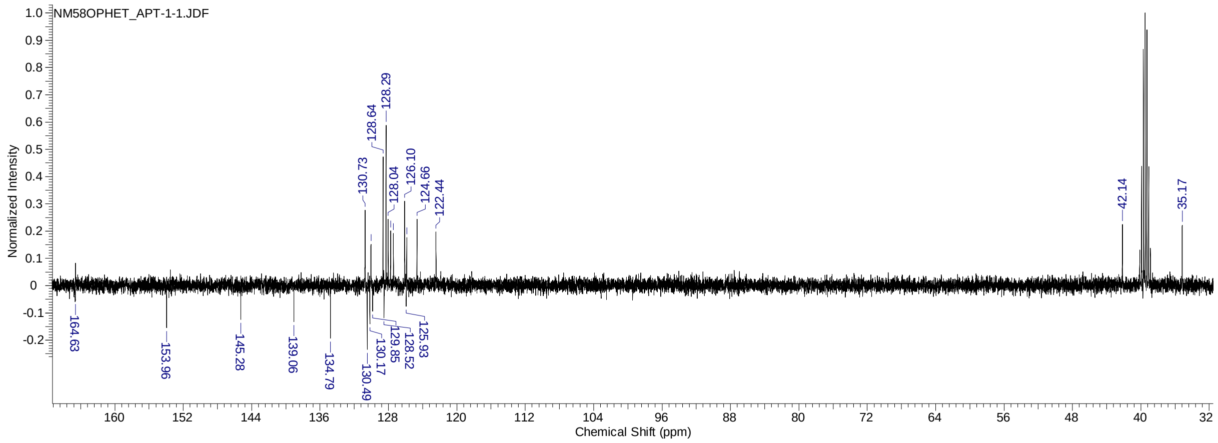

Figure S28. ^{13}C-NMR (DMSO-d_6) spectrum of 2-[*N*-(2,4,5-trichlorophenyl)carbamoyl]naphthalen-1-yl 2-phnylethyl carbamate (**13**).

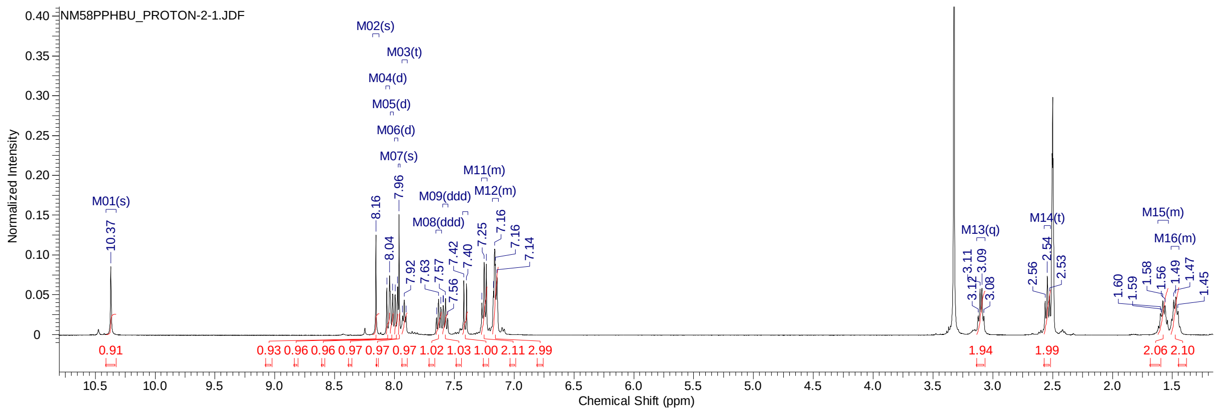

Figure S29. ^{1}H-NMR (DMSO-d_6) spectrum of 2-[*N*-(2,4,5-trichlorophenyl)carbamoyl]naphthalen-1-yl 4-phenylbutyl carbamate (**14**).

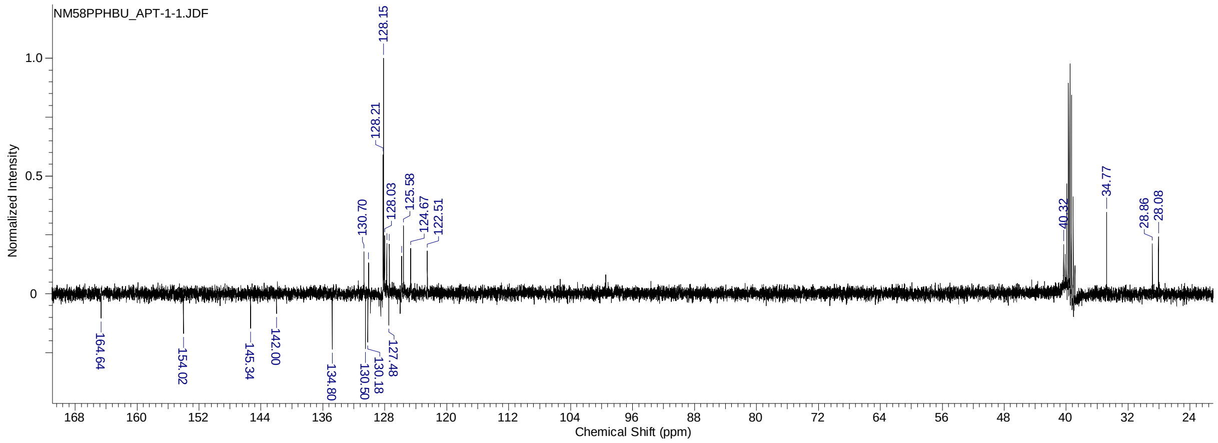

Figure S30. ^{13}C-NMR (DMSO-d_6) spectrum of 2-[*N*-(2,4,5-trichlorophenyl)carbamoyl]naphthalen-1-yl 2-phnylbutyl carbamate (**14**).

Determination of Stability

Carbamates are a relatively unstable part of the molecule, so it is useful to know the stability of such modified molecules. Ethyl carbamate **2** was selected for stability evaluation as the derivative with the expected lowest stability due to the least steric protection of the carbamate bond. The decomposition process of carbamate **2** at pH 6, 7 and 8 can be described by pseudo-first order kinetics. The calculated values of decomposition (reaction) rate and half-life are given in Table S1 and were calculated from the slope of the graphs of the natural logarithm of the residual reactant fraction as a function of time by statistical regression analysis. Table S1 shows that the ethyl derivative **2** at alkaline pH 8 has a $\tau_{0.5}$ of about 9 days, while already at neutral pH the $\tau_{0.5}$ is approx. 23 days. As biological experiments are performed in media with a pH range of 6-7 and a maximum of 48 hours, it can be stated that the compounds are stable enough to be tested for biological activity.

Approximately 2 mg of carbamate **2** was weighed into 25 mL Erlenmeyer flasks and dissolved in phosphate buffers with pH 6, 7 and 8. The flasks were coated with parafilm. The prepared samples were stirred at 37 °C in a water bath for 3 days, fractions were taken at time intervals (0, 0.5, 1, 1.5, 2, 2.5, 3, 4, 5, 6, 12, 24, 48 and 72 h) and injected into the HPLC system. Each experiment was repeated three times. Each sample was analyzed twice.
A HPLC separation module Agilent 1200 Series (Agilent Technologies, Santa Clara, CA, USA) equipped with a Dual Absorbance Detector (DAD SL G1315C, Agilent Technologies) was used to

evaluate of ethyl carbamate stability. A chromatographic column XTerra® Phenyl 3.5 µm, 100 × 4.6 mm (Waters Corp., Milford, MA, USA) was used. The HPLC separation process was monitored by ChemStation for LC 3D systems (Agilent Technologies). Isocratic elution by a mixture of acetonitrile (HPLC grade, 60.0%) and phosphate buffer, pH 2.5 (40%) as a mobile phase was used. The total flow of the column was 1.0 mL/min, injection 20 µL, the time of analysis was 8 min, column temperature 40 °C, and sample temperature 10 °C. The detection wavelength of 254 nm was chosen. The retention time (t_R) of carbamate **2** was 4.92 ± 0.05 min and of the pattern anilide 3.12 ± 0.05 min.

Table S1. Ethyl carbamate **2** decomposition rate and half-life values.

**pH**	**k [h^{-1}]**	**$\tau_{0,5}$ [h]**
6	5.77×10^{-4}	1202
7	1.25×10^{-3}	556
8	3.17×10^{-3}	219
